# Synthesis, and characterization of metallic glassy Cu–Zr–Ni powders decorated with big cube Zr_2_Ni nanoparticles for potential antibiofilm coating applications

**DOI:** 10.1038/s41598-022-17471-x

**Published:** 2022-08-01

**Authors:** Ahmad Aldhameer, M. Sherif El-Eskandarany, Mohmmad Banyan, Fahad Alajmi, Mohamed Kishk

**Affiliations:** 1 Biotechnology Program, Environment & Life Science Research Center, Kuwait Institute for Scientific Research, 13109 Kuwait, Kuwait; 2 Nanotechnology and Advanced Materials Program Energy and Building Research Center, Kuwait Institute for Scientific Research, 13109 Kuwait, Kuwait

**Keywords:** Materials science, Biomaterials

## Abstract

Biofilms, are significant component that contributes to the development of chronic infections, especially when medical devices are involved. This issue offers a huge challenge for the medical community since standard antibiotics are only capable of eradicating biofilms to a very limited degree. The prevention of biofilm formation have led to the development of a variety of coating methods and new materials. These methods are intended to coat surfaces in such a way as to inhibit the formation of biofilm. Metallic glassy alloys, in particular, alloys that include copper and titanium metals have gained popularity as desirable antibacterial coating. Meanwhile, there has been a rise in the use of the cold spray coating technique due to the fact that it is a proper approach for processing temperature-sensitive materials. The present study was carried out in part with the intention of developing a new antibiofilm metallic glassy consisting of ternary Cu–Zr–Ni using mechanical alloying technique. The spherical powders that comprised the end-product were utilized as feedstock materials for cold spray coatings to stainless steel surfaces at low temperature. When compared to stainless steel, substrates coated with metallic glassy were able to significantly reduce the formation of biofilm by at least one log.

## Introduction

The capacity of any society throughout human history to design and instigate the introduction of novel materials that meet their specific requirements has resulted in the improvement of their performance and ranking in the globalized economy^1^. It is always attributed to man's ability to develop materials and manufacturing equipment and devises used for materials fabrication and characterization, as measured by progress made in health, education, industry, economics, culture, and other areas, from one country or region to another, and this is true regardless of the country or region^2^. Materials scientists have devoted the considerable time over the 60 years focusing their attention on one primary concern: the pursuit for novel and cutting-edge materials. Recent research has concentrated on enhancing the qualities and performance of already existing materials, as well as synthesizing and inventing whole new types of materials.

The incorporation of alloying elements, the modification of the material's microstructure, and the application of thermal, mechanical, or thermo-mechanical processing techniques have led to significant enhancements in the mechanical, chemical, and physical properties of a variety of different materials. In addition, hitherto unheard-of compounds have been successfully synthesized at this point. These persistent efforts have led to the birth of new families of innovative materials that are collectively referred to as advanced materials^2^. Nanocrystalline, nanoparticles, nanotubes, quantum dots, zero dimensional, amorphous metallic glasses, and high entropy alloys are just some of the examples of advanced materials that were introduced to the worldwide since the middle of the past century^1^. When it comes to the fabrication and developing of new alloys with superior characteristics, it is often a question of increasing the deviation from equilibrium, in either the final product or at an intermediate stage of its production. As a result of the implementation of new preparation techniques for having a significant deviation from equilibrium, an entirely new class of metastable alloys called metallic glasses was discovered^3^.

### Metallic glasses

His work at the California Institute of Technology in 1960 brought about a revolution in the concept of metallic alloys when he synthesized an Au–25 at.% Si alloy in the glassy state by rapidly solidifying the liquid at rates approaching a million degrees per second^4^. Professor Pol Duwezs’ discover event not only heralds the beginning of the history of metallic glass (MG), but it also led in a paradigm change in the way people thought about metal alloys. Since the earliest pioneer investigation for synthesizing MG alloys, practically all metallic glasses are entirely produced through the use of one of the following methods; (i) rapid solidification of melts or vapors, (ii) atomic disordering of crystalline lattices, (iii) solid-state amorphization reaction between pure metallic elements, and (iv) solid-state transformations from metastable phases^5^.

MGs are distinguished by the fact that they lack the long-range atomic order associated with crystals, which is defining feature of crystals. In today's world, there has been tremendous progress in the area of metallic glasses. They are novel materials with intriguing properties that are of interest not only in solid-state physics but also in metallurgy, surface chemistry, technology, biology and many others. Such new class of materials exhibit characteristics that are significantly distinct from those of solid metals, making them intriguing candidates for technological applications in a variety of fields. They possess some of the important properties; (i) high mechanical ductility and yield strength, (ii) high magnetic permeability, (iii) low coercive forces, (iv) unusual corrosion resistance, (v) temperature-independent electrical conductivity^6,7^.

### Mechanical alloying

**Figure 1:**
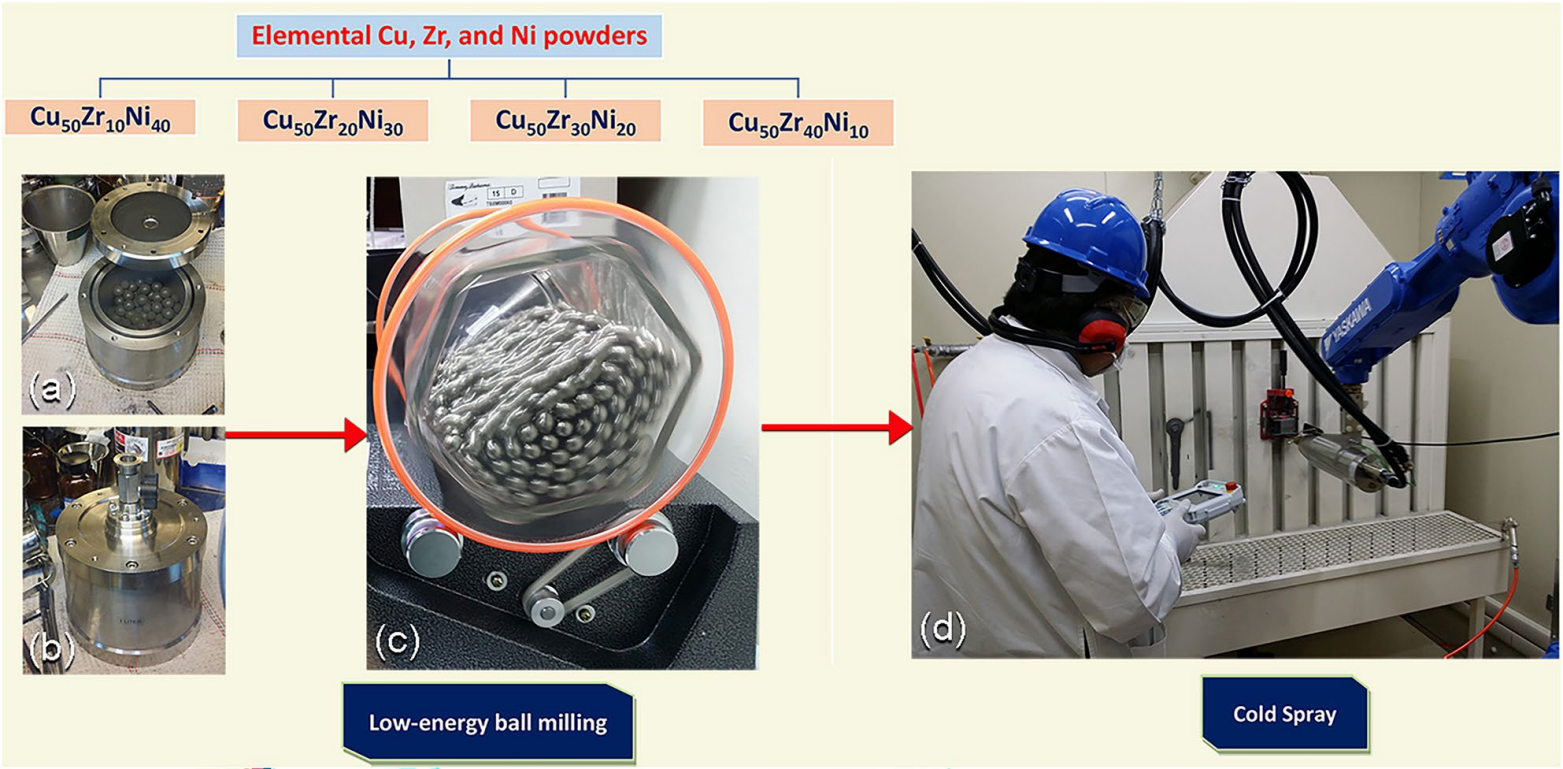
Schematic presentation displays the fabrication methods employed in the present study for preparation of Cu_50_(Zr_50−x_Ni_x_) metallic glass (MG) coated/SUS 304. (**a**) Preparations of MG alloy powders with different Ni concentration, x (x; 10, 20, 30, and 40 at.%) using low energy ball milling technique. (**a**) The starting materials were charged into tool steel vial together with tool steel balls, and (**b**) sealed inside a glove box filled with He atmosphere. (**c**) A transparent milling container model illustrated the balls movement during the milling process. The end-product of the powders obtained after 50 h were used to coat SUS 304 substrate, using cold spray approach (**d**).

Mechanical alloying (MA)^1,8^ is a relatively new technique that was first introduced by Professor C.C. Kock and his colleagues in 1983^9^. They prepared amorphous Ni_60_Nb_40_ powders by milling a mixture of pure elements at ambient temperature, which was very close to room temperature. In general, the MA reaction is carried out between the diffusion couplings of the reactant material powders in a reactor, which is usually made of stainless steel and referred to as a ball mill^10^ (Fig. 1a,b). Since then, this mechanically-induced solid-state reaction technique has been utilized to prepare novel families of amorphous -/metallic glassy alloy powders, using low- (Fig. 1c) and high energy ball mills, as well as rod-mills^11–16^. In particular, this approach has been used to prepare immiscible systems such as Cu–Ta^17^, and high melting point alloys for example Al-transition metal systems (TM; Zr, Hf, Nb, and Ta)^18,19^, and Fe–W^20^, which cannot be obtained using the conventional preparation routes. Additionally, MA has been considered as one of the most powerful nanotechnology tools for preparations industrial scale of nanocrystalline and nanocomposite powder particles^21^ of metal-oxides, -carbides, -nitrides, -hydrides, carbon nanotubes, nanodiamonds, as well as wide range of stable and metastable phases via top-down approach^1^.

### Surface protective coating

When it comes to bulk materials surfaces (substrates), surface engineering is concerned with the design and modification of the surfaces (substrates) in order to give certain physical, chemical, and technical qualities that were not intrinsically included in the original bulk materials^22^. Some of the features that may be effectively improved by surface treatments include wear, oxidation, and corrosion resistance, friction coefficients, bio-inertness, electrical properties, and thermal insulation, to name a few examples^1^. Improvements in surface qualities may be produced by the use of metallurgical, mechanical, or chemical techniques. As a well-known process, coating is simply defined as a single or multilayered materials deposited artificially on the surface of a bulk object (substrate) made of another material. Hence, coating is used to obtain some required technical or decorative properties in part, as well as to protect the material from expected chemical and physical interactions with its surrounding environment^23^.

For depositing the appropriate surface protective layer with a thickness ranging from a few micrometers (below 10–20 µm) to more than 30 µm and even several millimeters, many methodologies and technologies may be applied. Overall, coating processes can be divided into two categories: (i) wet coating approaches, which include electroplating, electroless plating, and hot-dip galvanizing methods, and (ii) dry coating approaches, which include brazing, weld overlays, physical vapor deposition (PVD), chemical vapor deposition (CVD), thermal spray techniques, and most recently cold spray technique^24^ (Fig. 1d).

### Antibiofilm protective coating

Biofilm is defined as a microbial community irreversibly attached to a surface and surrounded in self-producing extracellular polymeric substances (EPS). Mature biofilm formation on surfaces can results in a major loss in many industrial sectors include food industry, water systems and health care environments. In human, more than 80% of microbial infections cases including Enterobacteriaceae and Staphylococcaceae species are very challenging to treat when biofilms formed. Moreover, it has been reported that in comparison to planktonic bacterial cells mature biofilm can be 1000-fold more resistant to the antibiotic treatment which considered as major therapeutic challenges. Antibacterial surface coating materials derived from conventional organic compounds have historically been employed. Although such materials often include toxic components that are potentially risky for humans^25,26^, it may help to avoid both the spread of bacteria and the destruction of substances.

The widespread bacterial resistance to antibiotic treatments because of biofilm formation led to the need to develop an effective antibiofilm coated surfaces that can be safe to apply^27^. Developing an anti-adhesive surface, both physically or chemically, that inhibit bacteria cells from bond to it and building biofilms as a consequence of the adhesion is the first approach in the process^27^. Developing coatings that enable antimicrobial chemicals to be given in highly concentrated and tailored amounts precisely where they are needed is the second technique. Achieving this by the development of unique coating materials such as graphene/germanium^28^, black diamond^29^ and ZnO-doped diamond-like carbon coatings^30^ that are bacterial resistant, such technique can minimize the toxicity and resistance development occurring because of biofilm formation^31^. Furthermore, coating in which bactericidal chemicals are bonded to the surface in order to give long-term protection against bacterial contamination^32^, is becoming more popular. Although all three procedures are capable of imparting an antimicrobial effect on the coated surface, they each have their own set of limitations that should be taken into consideration when establishing a strategy for application.

### Present status of antimicrobial coating materials

Products that are currently on the market has been hindered by the fact that insufficient time has been devoted to the analysis and testing of the bioactive components that are contained in the protective coating^33–35^. Companies are making claims that their products would provide users with the ideal functional aspects; however, this has been a barrier to the success of the products that are currently on the market. Compounds derived from silver are used in the great majority of antimicrobial therapies that are now available to consumers. These goods have been developed to protect users against the potentially hazardous effects of microbes. The delayed antibacterial effect and associated toxicity of silver compounds have increased the amount of pressure placed on researchers to develop an alternative that is less hazardous^36,37^. The creation of a worldwide antimicrobial coating that is suitable for use both inside and outside is still proving to be a difficult task. This is due to the fact that there are associated dangers to both health and safety. Discovering an antibacterial agent that is less harmful to humans and finding out how to include it into a coating matrix that has a longer shelf life is a goal that is tremendously sought after^38^. The most recent antimicrobial and antibiofilm materials aim to kill bacteria either on direct contact or in close proximity after the release of the active agent. They can do so by either inhibiting the initial bacterial adhesion, which involves counteracting the formation of a protein layer on the surface, or by killing bacteria by disturbance of cell wall^35^.

### Cold spraying technique

**Figure 2:**
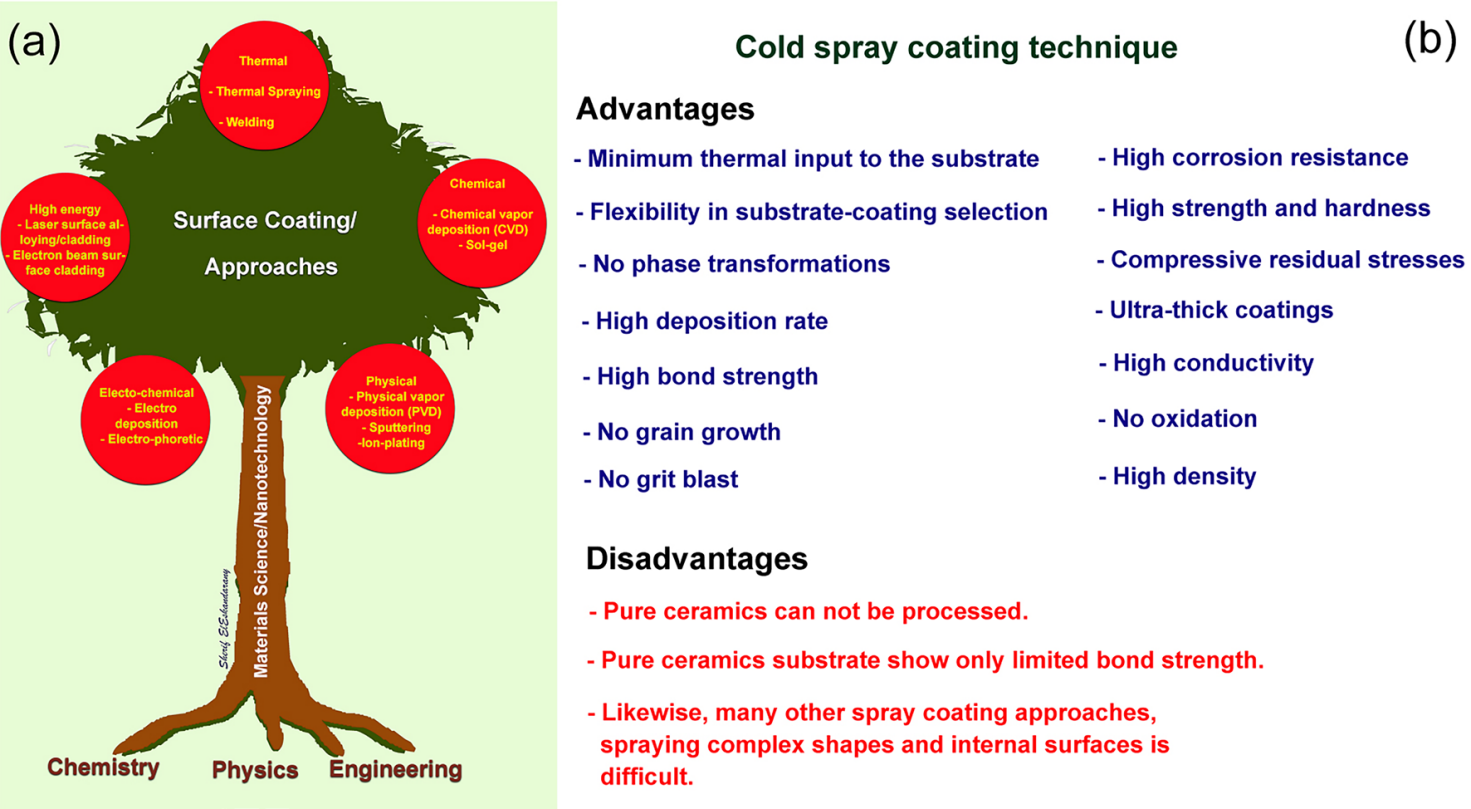
(**a**) Illustration displays the major fabrication technology used for surface, and (**b**) selected advantages and disadvantages of cold spray coating technique.

Fundamentally, surface coating is the process of placing another layer to a component's surface in order to enhance surface-dependent qualities. The goal of surface coating is to customize the microstructure and/or composition of the near-surface area of the component^39^. The surface coating technology can be classified into different approached that are summarized in Fig. 2a. Coating may be subdivided into the categories of thermal, chemical, physical, and electrochemical, depending on the method that was used to create it.

Cold spray technique has many parallels to regular thermal spray methods. However, there are also major basic characteristics that make the cold spray process and cold-sprayed materials particularly distinctive. The cold spray technology is still in its infancy, but it shows a great deal of promise. In certain applications, the unique properties of cold spray provide substantial benefits, over-coming inherent limits of typical thermal spray method. It provides a means of overcoming significant constraints of conventional thermal spray technologies, in which the powders must be melt during the thermal spray technique in order to be deposited onto the substrate. Obviously, this traditional coating process is not suitable with the materials that are very sensitive to temperature, such as nanocrystalline, nanoparticles, amorphous, and metallic glasses^40–42^. Moreover, the as- thermal spray coating material are always show high levels of porosity and oxide. In contrast to the thermal spray technique, cold spray technique possesses many significant advantages, such as (i) minimum thermal input to the substrate, (ii) flexibility in substrate-coating selection, (iii) absence of phase transformations, and grain growth, and (iv) high bond strength^1,39^ (Fig. 2b). In addition, the as—cold—spray coating material reveal high corrosion resistance, high strength and hardness, high conductivity, and high density^41^. Contrary to the advantages of cold spray coating process, there are still few disadvantages upon using this technique, as listed in Fig. 2b. When coating with pure ceramics powders such as Al_2_O_3_, TiO_2_, ZrO_2_, and WC, the cold spray method cannot be used. Ceramics/metal composite powders, on the other hand, may be used as feedstock materials for coatings. The same is true for the other approaches of thermal spraying; it is still difficult to spray complicated surfaces and the inside surfaces of pipes.

In light of the objective of the current work, which is to use metallic glassy powders as feedstock coating materials, it is obvious that traditional thermal spray cannot be used for such a purpose. This is due to the fact that the metallic glassy powders will crystallize upon the application of high temperatures^1^.

### Aim of the present work

The bulk of the tools used in the medical and food sectors are made of austenitic stainless steel alloys (SUS316 and SUS304), which have a high chromium content ranging between 12 and 20 wt% and are utilized in the production of surgical instruments. It is understood that using chromium metal as an alloying element in steel alloys may greatly increase the corrosion resistance of a standard steel alloy, and this is commonly accepted. Stainless steel alloys, despite their high corrosion resistance, do not exhibit substantial antibacterial characteristics^38,39^. This is in contrast to their high corrosion resistance. Following this, it is possible to anticipate the development of infection and inflammation, which are mostly caused by the adhesion and colonization of bacteria on the surfaces of stainless steel biomaterials. With a significant difficulty linked with the bacterial adhesion and biofilm formation pathways, significant difficulties may arise, which may result in a deterioration in health, which may have a number of consequences that might directly or indirectly impact human health.

The present study is the Phase I of a project funded by the Kuwait Foundation for the Advancement of Sciences (KFAS), under Contract number: 2010-550401 to investigate the feasibility of using MA technique for production of metallic glassy Cu–Zr–Ni ternary powders (Table 1), for the purpose of producing antibiofilm/SUS304 surface protective coating. The Phase II of the project, which will be started on January 2023, will examine in detail the electrochemical corrosion characteristics and mechanical behavior of this system. Detailed microbiological testing of different bacterial species will be conducted.

**Table 1:** Elemental analysis conducted by field-emission scanning electron microscope (FE-SEM)/energy dispersive X-ray spectroscopy (EDS) of the starting materials for as-hand mixed Cu_50_Zr_40_Ni_10_, Cu_50_Zr_30_Ni_20_, Cu_50_Zr_20_Ni_30_, and Cu_50_Zr_10_Ni_40_ powders. All of the metallic glassy Cu_50_(Zr_50−x_Ni_x_) alloys listed in Table were prepared by mechanical alloying (MA) method with the use of a low-energy ball mill (Fig. 1a).

System	Chemical composition (wt%)		
	Cu	Zr	Ni
Cu_50_Zr_40_Ni_10_	42.86	49.22	7.92
Cu_50_Zr_30_Ni_20_	44.83	38.61	16.58
Cu_50_Zr_20_Ni_30_	46.98	26.98	26.04
Cu_50_Zr_10_Ni_40_	49.36	14.17	36.47

In the present paper, the influence of the Zr alloying element content on the glass forming ability (GFA) is discussed based on morphological, and structural characteristics. Aside from that, the antibacterial properties of coated metallic glassy powders coated/SUS304 composite is explored. In addition, the present work has been carried out to investigate the possibility of structural transformations of metallic glassy powders occurring during the cold spray process within the supercooled liquid region of the fabricated metallic glassy system. As representative examples, the Cu_50_Zr_30_Ni_20_ and Cu_50_Zr_20_Ni_30_ metal glassy alloys have been used in this investigation.

## Results and discussion

### Morphology

**Figure 3:**
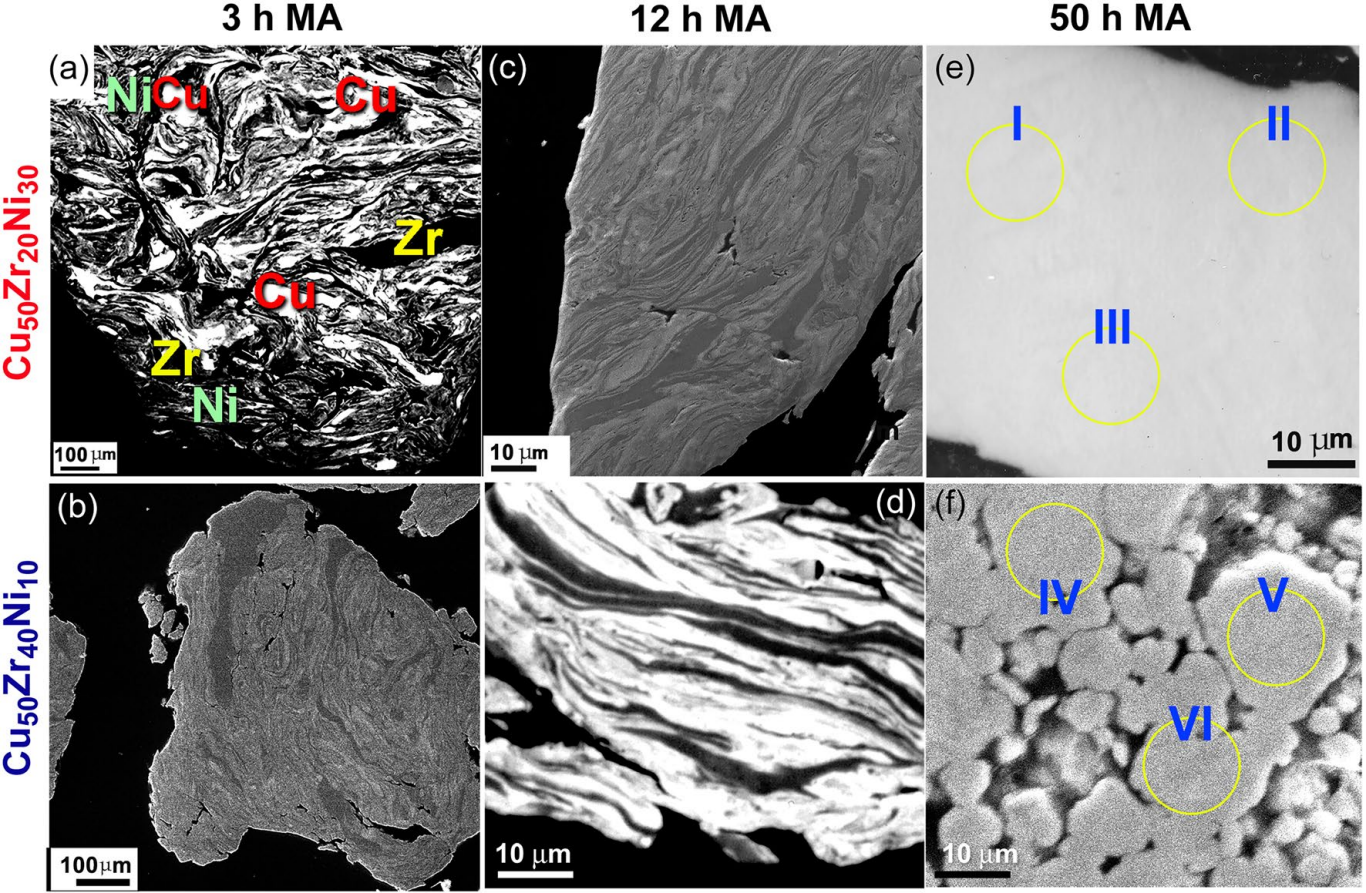
Metallographic characteristics of mechanically alloyed (MA) powders obtained after different stages of ball milling time. The field-emission scanning electron microscope (FE-SEM) images of MA, and Cu_50_Zr_40_Ni_10_ powders obtained after 3, 12, and 50 h of low energy ball milling time, are presented in (**a**), (**c**), and (**e**) for Cu_50_Zr_20_Ni_30_ system, whereas the corresponding images of the Cu_50_Zr_40_Ni_10_ system, taken after the same MA time are displayed in (**b**), (**d**), and (**f**).

In this section, the morphological changes that were taken place upon low-energy ball milling of elemental Cu, Zr, and Ni powders are presented. As illustrative instances, two different systems composed of Cu_50_Zr_20_Ni_30_, and Cu_50_Zr_40_Ni_10_ will be used as reprehensive examples. The MA process may be divided into three distinct stages, as indicated by the metallographic characteristics of the powders produced during the milling stages (Fig. 3).

During the ball milling process, the amount of effective kinetic energy that could be delivered to the metal powders was affected by a combination of parameters, as illustrated in Fig. 1a. This includes collision between both the balls and the powders, pressure clipping of powders stuck between milling media or between the milling media and the, impact of the falling balls, shear and abrasion caused by dragging of powders between moving ball milling media, and shock wave transmitted through crop load by falling balls (Fig. 1a). As a result of cold-welding taking place during the early stage of MA (3 h), elemental Cu, Zr, and Ni powders were heavily deformed to produce large powder particles (larger than 1 mm in diameter). These large composite particles are characterized by formation of thick lamella of the alloying elements (Cu, Zr, Ni), as presented in Fig. 3a,b. Increasing the MA time to 12 h (intermediate stage), led to increase the kinetic energy of the ball mill, leading to disintegrate the composite powders into finer powders (less than 200 µm), as shown in Fig. 3c,d. At this stage, the applied shear forces lead to the formation of fresh-metallic surfaces, having fine intimated layers of Cu, Zr, Ni, as displayed in Fig. 3c,d. As a result of layer refining, a solid-stage reaction was taken place at the interfaces of the lamella to produce a new phase.

At the culmination of the MA process (after 50 h), the lamella-like metallography was just faintly visible (Fig. 3e,f), but the polished surface of the powders displayed mirror-like metallography. This signifies that the MA process has been completed and the production of a single reacting phase has taken place. The elemental composition of the zones indexed in Fig. 3e (I, II, III),f was determined by using the field-emission scanning electron microscope (FE-SEM) in conjunction with energy dispersive X-ray spectroscopy (EDS) (IV, V, VI).

In Table 2, the elemental concentration of the alloying elements is shown as a percentage of the total weight for each of the zones that were chosen in Fig. 3e,f. When these results are compared to those of the starting nominal composition for Cu_50_Zr_20_Ni_30_ and Cu_50_Zr_40_Ni_10_, which are listed in Table 1, it is possible to realize that the composition of these two end products had values that were extremely similar to the nominal composition. Additionally, the associated composition values for the zones listed in Fig. 3e,f did not refer to a significant deterioration or fluctuation in composition from one zone to another for each sample. This is evidenced by the fact that there is no change in composition from one zone to another. This points to the production of homogenous alloy powders, as shown in Table 2.

**Table 2:** Elemental analysis conducted by FE-SEM in conjunction with EDS for as prepared Cu_50_Zr_20_Ni_30_, and Cu_50_Zr_40_Ni_10_ alloy powders obtained after 50 h of mechanical alloying (MA) time. Whereas Zones I, II, and III are related to the indexed circular symbols in Fig. 3e, Zones IV, V, and VI refer to the zones presented in Fig. 3f.

	Cu_50_Zr_20_Ni_30_			Cu_50_Zr_40_Ni_10_		
	Elemental composition (wt%)					
	Cu	Zr	Ni	Cu	Zr	Ni
Zone I	46.83	27.24	25.93	–	–	
Zone II	46.86	27.21	25.93	–	–	
Zone III	46.82	27.09	26.09	–	–	
Zone IV	–	–	–	42.86	49.26	7.88
Zone V	–	–	–	42.81	49.25	7.94
Zone VI	–	–	–	42.78	49.14	7.98

**Figure 4:**
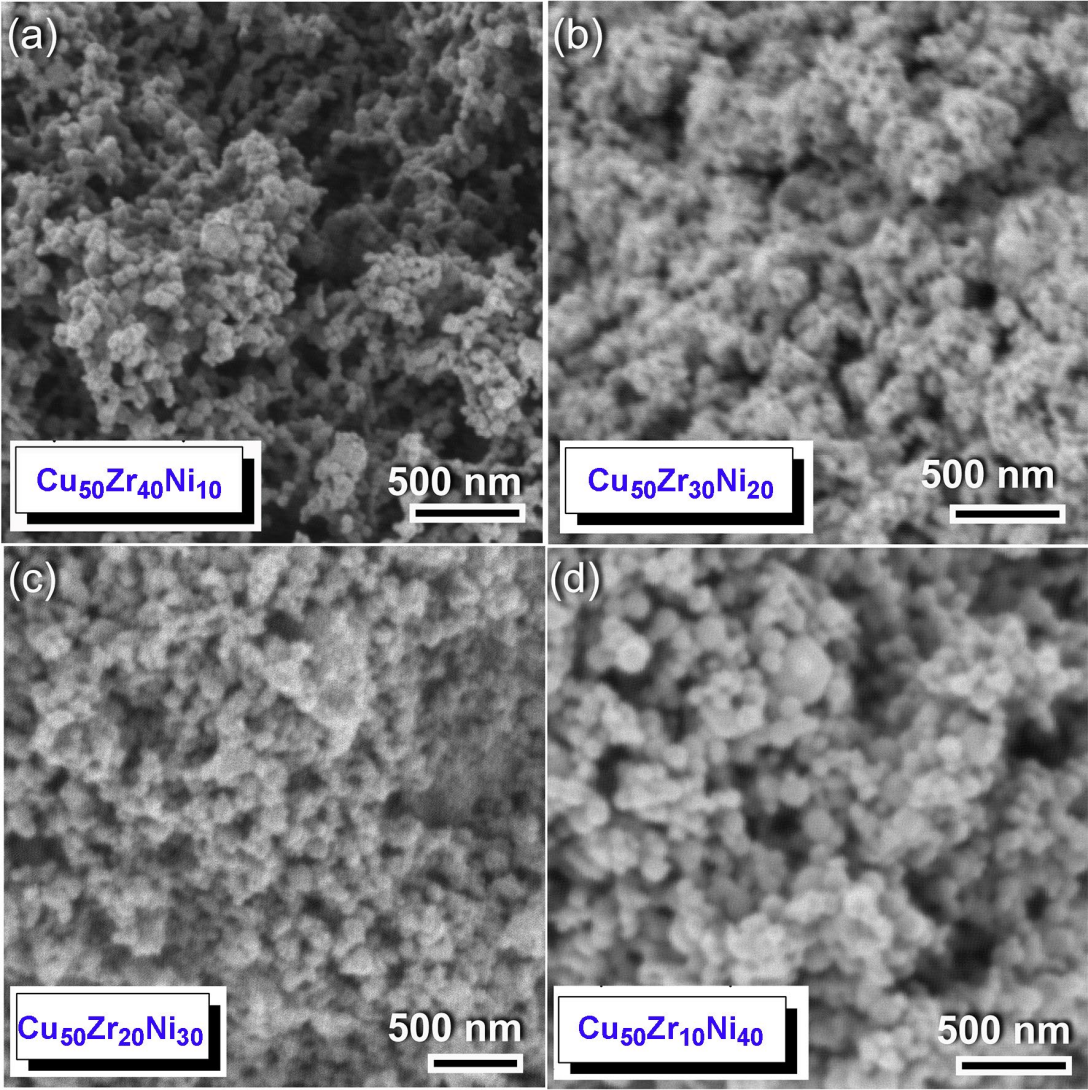
Morphological characteristics of Cu_50_(Zr_50−x_Ni_x_) powders obtained after 50 h of MA time. The FE-SEM images of the powders obtained after 50 of MA time are shown in (**a**), (**b**), (**c**), and (**d**) for Cu_50_Zr_40_Ni_10_, Cu_50_Zr_30_Ni_20_, Cu_50_Zr_20_Ni_30_, Cu_50_Zr_10_Ni_40_ systems, respectively.

FE-SEM micrographs for the end-product Cu_50_(Zr_50−x_Ni_x_) powders, obtained after 50 of MA time, is presented in Fig. 4a-d for x equals 10, 20, 30 and 40 at.%, respectively. Following this step of milling, the powders were aggregated due to the van der Walls effect, which, resulted in the formation of large aggregates consisting of ultrafine particles with diameters ranging from 73 to 126 nm, as seen in Fig. 4.

**Figure 5:**
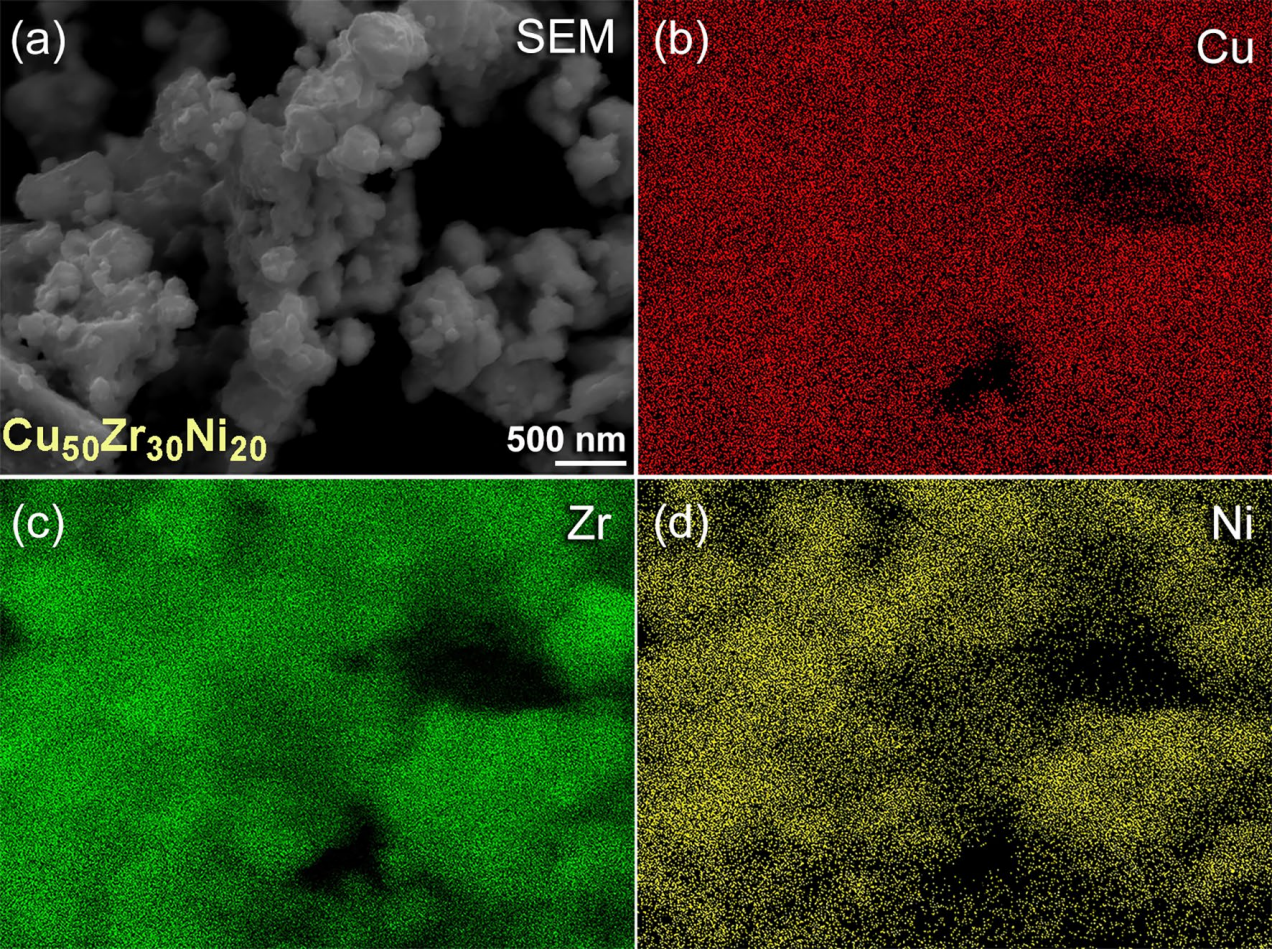
Morphological and local elemental distributions conducted by FE-SEM/energy dispersive X-ray spectroscopy (EDS) for MG Cu_50_Zr_30_Ni_20_ powders obtained after 50 of MA time. (**a**) SEM, and X-ray EDS mapping of (**b**) Cu-K_α_, (**c**) Zr-L_α_, and (**d**) Ni-K_α_ images.

Before the powders were charged into the cold spray feeder, they were first sonicated for fifteen minutes in ethanol of analytical grade, and then they were dried at a temperature of 150 °C for two hours. This step had to be taken in order to successfully combat the agglomeration that often caused a number of significant issues throughout the coating process^1^. After the completion of the MA process, further characterizations were carried out in order to investigate into the degree to which the alloy powders were homogenous. Figure 5a–d, respectively, illustrate the FE-SEM micrograph as well as the corresponding EDS maps for the alloying elements of Cu, Zr, and Ni of Cu_50_Zr_30_Ni_20_ alloy obtained after 50 h of M time. To point out the obvious, the alloy powders that were produced after this step were uniform since they did not reveal any compositional fluctuations that went beyond the sub-nano level, as shown in Fig. 5.

### General crystal structure

**Figure 6:**
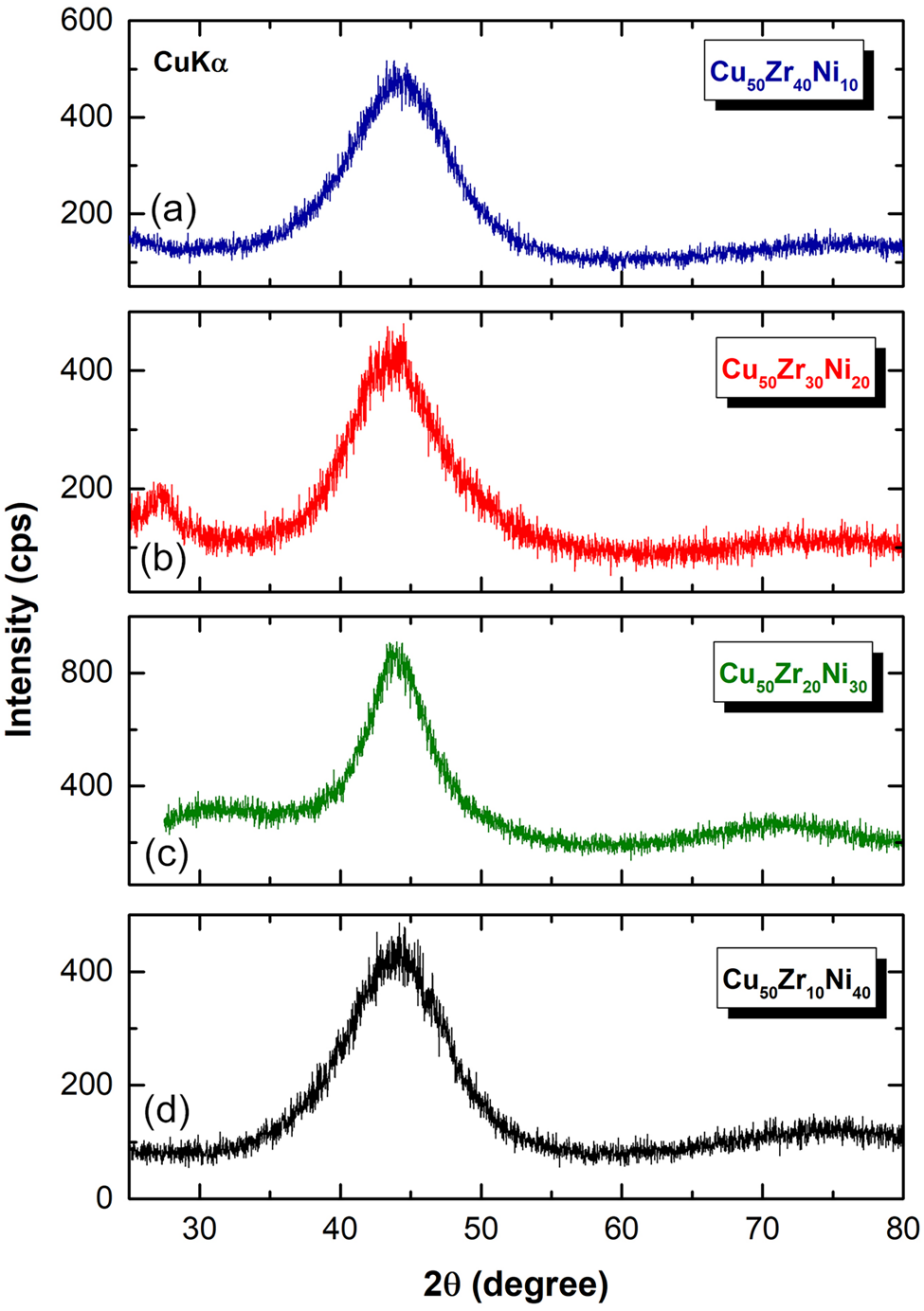
XRD patterns of (**a**) Cu_50_Zr_40_Ni_10_, (**b**) Cu_50_Zr_30_Ni_20_, (**c**) Cu_50_Zr_20_Ni_30_, and (**d**) Cu_50_Zr_20_Ni_30_ powders obtained after 50 h of MA time. All the samples without exceptions revealed halo-diffuse patterns, implying the formation of amorphous phase.

The XRD patterns of mechanically alloyed Cu_50_Zr_40_Ni_10_, Cu_50_Zr_30_Ni_20_, Cu_50_Zr_20_Ni_30_, and Cu_50_Zr_20_Ni_30_ powders obtained after 50 h of MA time are shown in Fig. 6a–d, respectively. After this stage of milling all the samples with different Zr concentrations have revealed amorphous structure, as characterized halo‐diffuse patterns that are displayed in Fig. 6.

### Local structure

**Figure 7:**
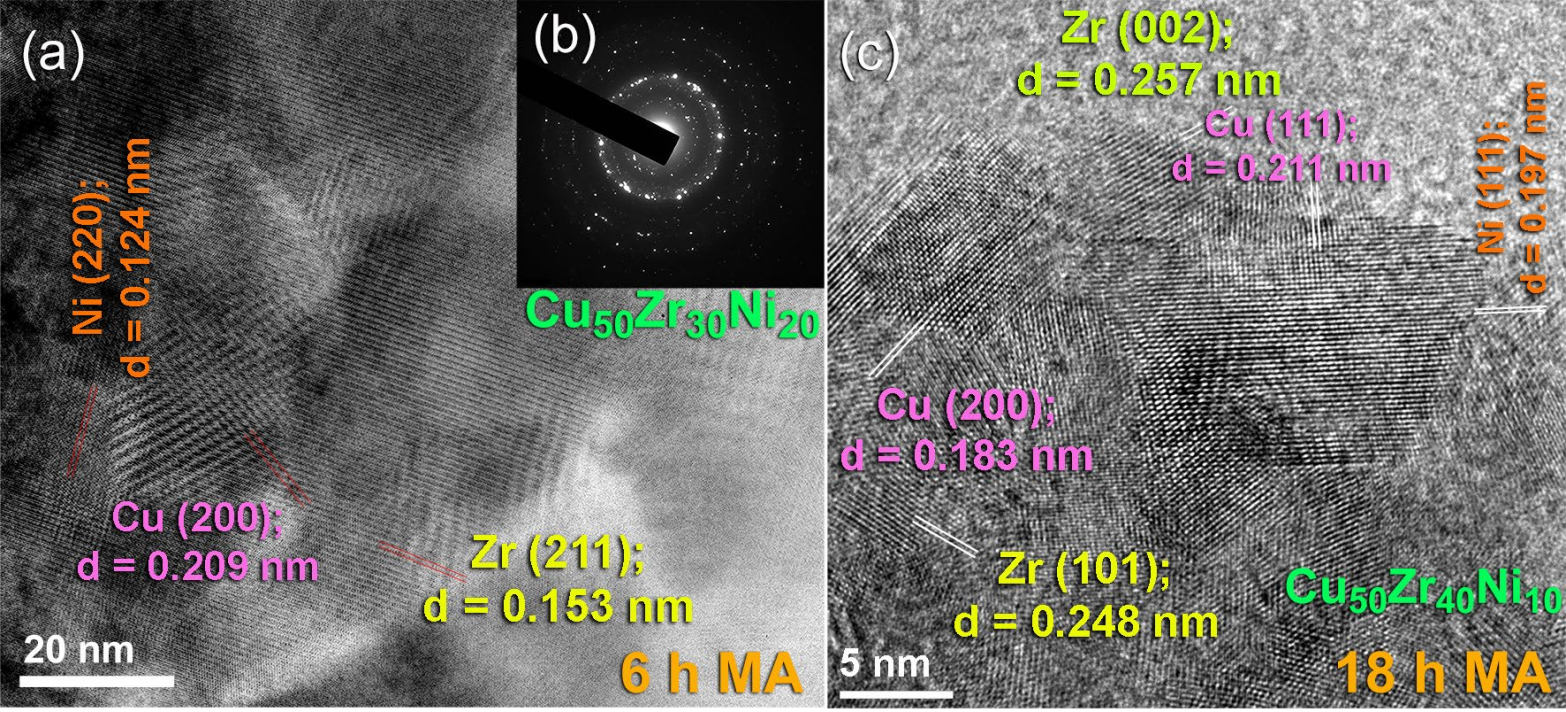
Local structure characteristics of the MA powders obtained after the early (6 h), and intermediate (18 h) stages. (**a**) Field-emission high resolution transmission electron microscope (FE-HRTEM), and (**b**) corresponding selected area diffraction pattern (SADP) of Cu_50_Zr_30_Ni_20_ powders after MA for 6 h. The FE-HRTEM image of Cu_50_Zr_40_Ni_10_ obtained after 18 h of MA time is displayed in (**c**).

The field-emission high-resolution transmission electron microscope (FE-HRTEM) was utilized in order to observe the structural changes and to comprehend the local structure of the powders that were produced as a consequence of ball milling for differing MA time. The FE-HRTEM images of powders obtained after the early (6 h), and intermediate (18 h) stage of milling for Cu_50_Zr_30_Ni_20_, and Cu_50_Zr_40_Ni_10_ powders are displayed in Fig. 7a,c, respectively. According to the bright field image (BFI) of the powders that were produced after 6 h of MA, the powders comprised of large grains with sharp boundaries of elemental fcc-Cu, hcp-Zr, and fcc-Ni, and there was no indication that a reacted phase had formed, as shown in Fig. 7a. Additionally, the related selected area diffraction pattern (SADP) taken from the middle zone of (a) revealed sharp-spot diffraction patters (Fig. 7b) indicating the existence of large crystallites and the absence of a reacted phase.

As can be seen in Fig. 7c, extending the MA duration to 18 h led to the development of severe lattice defects that were coupled with plastic deformation. During this intermediate stage of the MA process, the powders experienced from a variety of defects, including stacking faults, lattice defects, and point defects (Fig. 7). These defects led the large grains to split apart along their grain boundaries into subgrains that were less than 20 nm in size (Fig. 7c).

**Figure 8:**
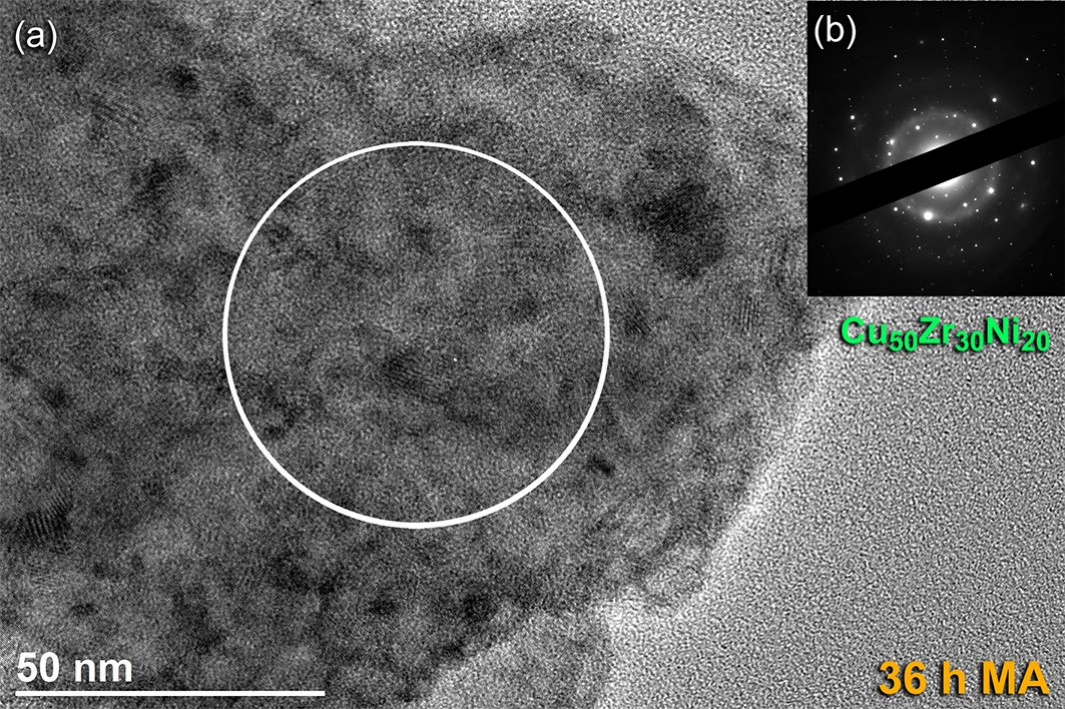
Local structural characteristics beyond the nano-level of 36 h-Cu_50_Zr_30_Ni_20_ powders. (**a**) Bright field image (BFI), and corresponding (**b**) SADP of Cu_50_Zr_30_Ni_20_ powders obtained after milling for 36 h of MA time.

The local structure of the Cu_50_Z_30_Ni_20_ powders that were milled for 36 h of MA time, possessed the formation of ultrafine nano grains embedded into a noncrystalline fine matrix, as shown in Fig. 8a. The local EDS analysis indicate that those nanocrystalline clusters shown in Fig. 8a were related to unprocessed alloying elements of Cu, Zr, and Ni powders. Meanwhile, the Cu content of the matrix fluctuated from ~ 32 at.% (poor region) to ~ 74 at.% (rich region), suggested the formation of heterogeneous product. Furthermore, the corresponding SADP of the powders obtained after this stage of milling, revealed halo diffuse primary and secondary rings of an amorphous phase, overlapped with sharp spots related to those unprocessed alloying elements, as displayed in Fig. 8b.

**Figure 9:**
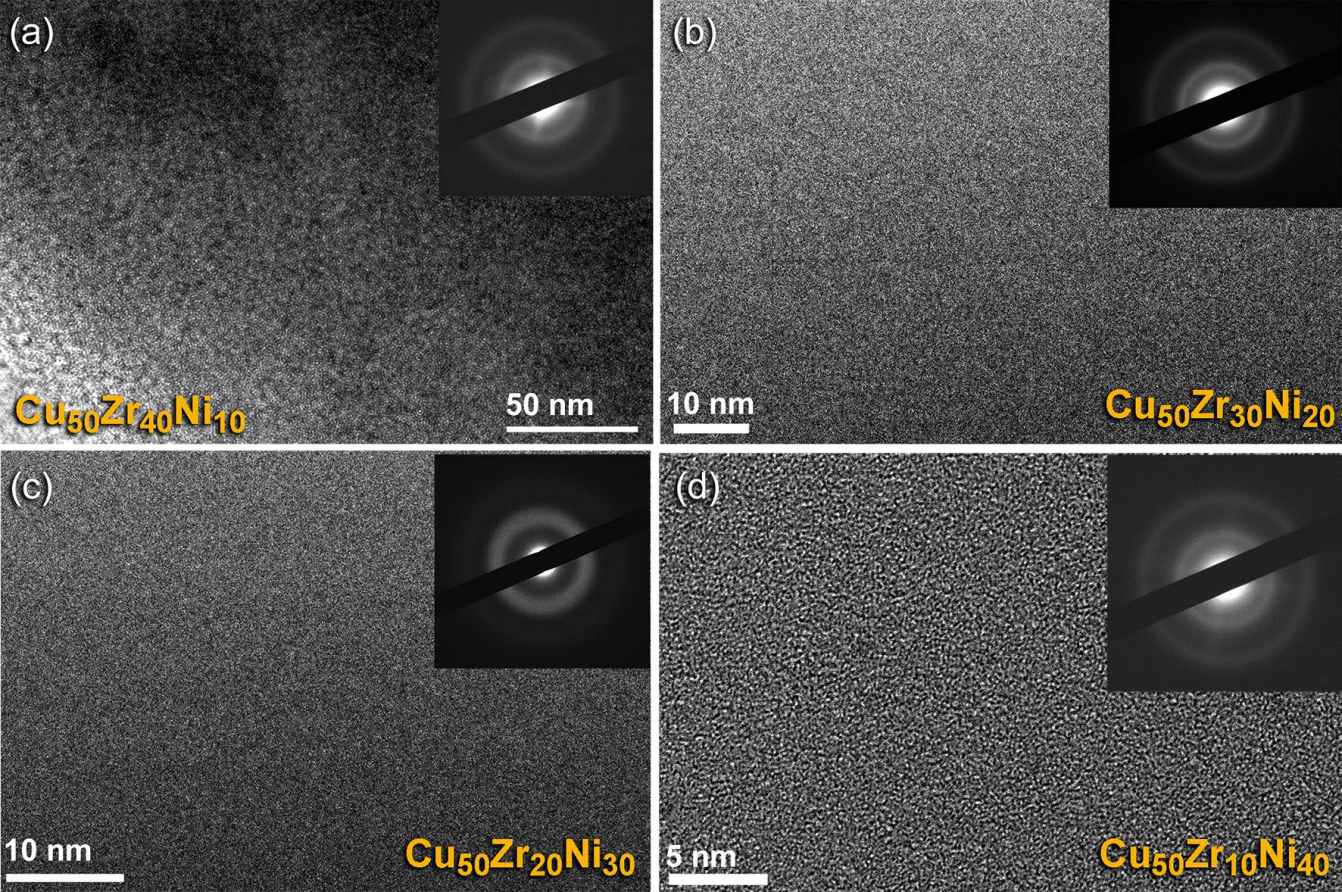
Local structure of the final product of MG Cu_50_(Zr_50−x_Ni_x_) systems. FE-HRTEM, and related nanobeam diffraction patterns (NBDPs) of (**a**) Cu_50_Zr_40_Ni_10_, (**b**) Cu_50_Zr_30_Ni_20_, (**c**) Cu_50_Zr_20_Ni_30_, and (**d**) Cu_50_Zr_10_Ni_40_ obtained after 50 h of MA time.

Toward the end of MA process (50 h), Cu_50_(Zr_50−x_Ni_x_), X; 10, 20, 30, and 40 at.% powders without any exceptions possessed maze-like morphology of amorphous phase, as displayed in Fig. 9a–d. In the corresponding SADP of each composition, neither spot-like diffraction nor sharp ring patterns can be detected. This indicates that unprocessed crystalline metals are not present, and instead, noncrystalline alloy powders have formed. These related SADPs that displayed halo diffuse patterns were also utilized as evidence of the development of an amorphous phase in the materials of the end-product.

### Thermal stability

**Figure 10:**
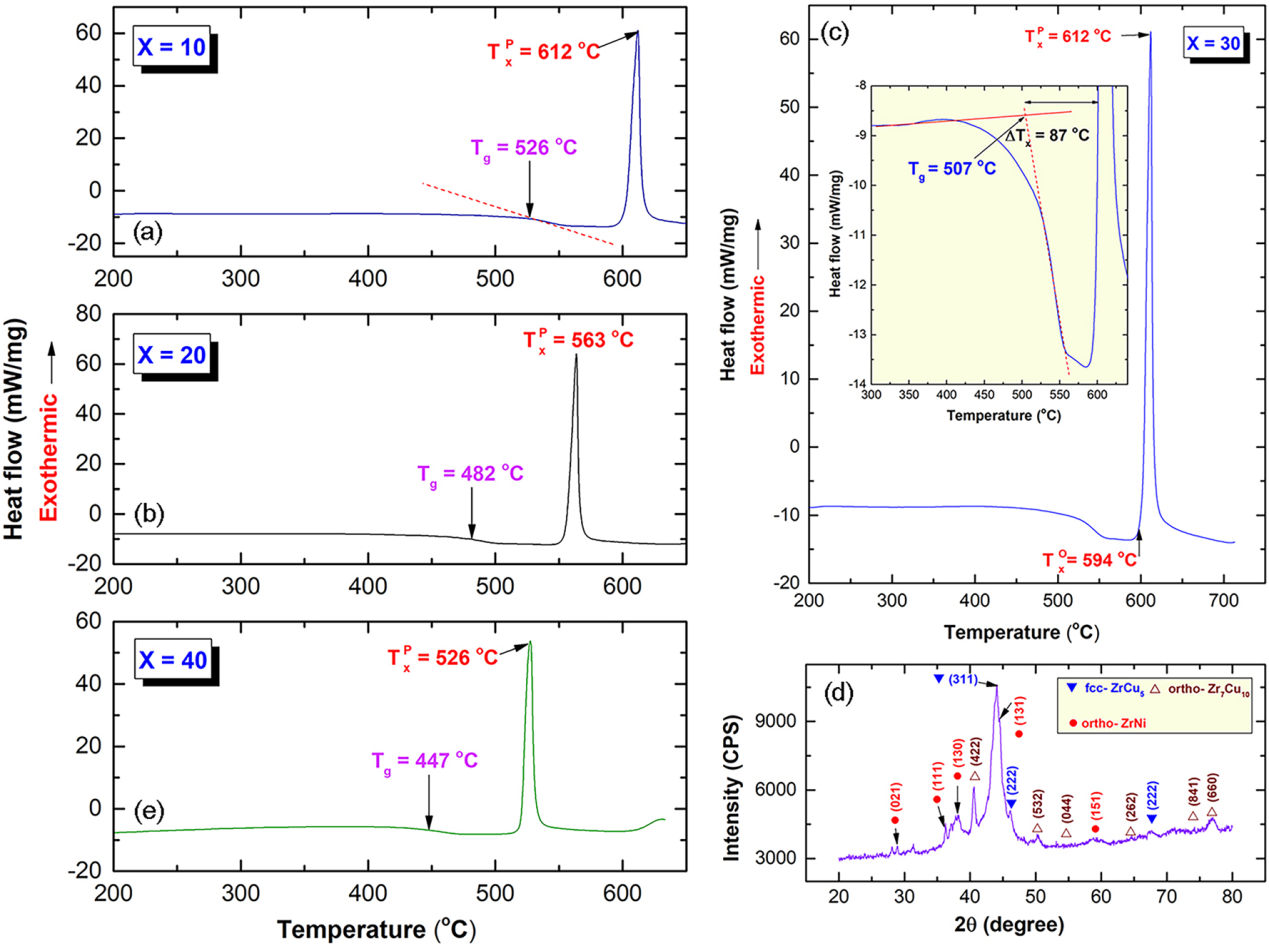
Thermal stabilities, indexed by glass transition temperature (T_g_), crystallization temperature (T_x_), supercooled liquid region (ΔT_x_) of Cu_50_(Zr_50−x_Ni_x_) MG powders obtained after 50 h of MA time. Differential scanning calorimeter (DSC) thermograms of (**a**) Cu_50_Zr_40_Ni_10_, (**b**) Cu_50_Zr_30_Ni_20_, (**c**) Cu_50_Zr_20_Ni_30_, and (**e**) Cu_50_Zr_10_Ni_40_ MG alloys powders obtained after 50 h of MA time. The x-ray diffraction (XRD) pattern of Cu_50_Zr_30_Ni_20_ sample that was heated in the DSC up to ~ 700 °C is shown in (**d**).

The thermal stability indexed by glass transition temperature (T_g_), supercooled liquid region (ΔT_x_), and crystallization temperature (T_x_) of amorphous Cu_50_(Zr_50−x_Ni_x_) systems, have been investigated as a function of Ni content (x) using differential scanning calorimetry (DSC) under flow of He gas. The DSC traces of Cu_50_Zr_40_Ni_10_, Cu_50_Zr_30_Ni_20_, and Cu_50_Zr_10_Ni_40_ amorphous alloy powders obtained after 50 h of MA time are presented together in Fig. 10a,b,e, respectively. Whereas the DSC trace for amorphous Cu_50_Zr_20_Ni_30_ is displayed individually in Fig. 10c. Meanwhile, of Cu_50_Zr_30_Ni_20_ sample that was heated in the DSC up to ~ 700 °C is shown in Fig. 10d.

As can be seen in Fig. 10, the DSC curves for all composition with different Ni concentrations (x) indicated two distinct occurrences, one endothermic and the other exothermic, respectively. The first events that are endothermic correspond to T_g_, whereas the second occurrences are correlated to T_x_. The region of span that existed horizontally between The horizontal span existed between T_g_ and T_x_ is referred to as the supercooled liquid region (ΔT_x_ = T_x_–T_g_). The results have indicated that T_g_, and T_x_ for Cu_50_Zr_40_Ni_10_ sample (Fig. 10a) that are laid at 526 °C, and 612 °C, respectively shifted to the low temperature side of 482 °C, and 563 °C upon increasing the Ni content (x) to 20 at.%, as displayed in Fig. 10b. Accordingly, the ΔT_x_ for Cu_50_Zr_40_Ni_10_ was decreased from 86 °C (Fig. 10a) to 81 °C for Cu_50_Zr_30_Ni_20_ (Fig. 10b). Decreasing the values of T_g_, T_x_, and ΔT_x_ to the level of 447 °C, 526 °C, and 79 °C was also observed for MG Cu_50_Zr_40_Ni_10_ alloy (Fig. 10b). This suggests that an increase in the Ni content led to a reduction in the thermal stability of the MG alloy. In contrast, MG Cu_50_Zr_20_Ni_30_ alloy exhibited a lower value of T_g_ (507 °C) in comparison to MG Cu_50_Zr_40_Ni_10_ alloy; nonetheless, its T_x_ showed a value that was comparable to the former (612 °C). As a consequence of this, ΔT_x_ exhibited a higher value (87 °C), as can be seen in Fig. 10c.

MG Cu_50_(Zr_50−x_Ni_x_) systems, exemplified by MG Cu_50_Zr_20_Ni_30_ alloy crystallized into crystalline phases of fcc-ZrCu_5_, orthorhombic-Zr_7_Cu_10_, and orthorhombic-ZrNi, through a single sharp exothermic peak (Fig. 10c). This noncrystalline to crystalline phase transformation was confirmed by the XRD (Fig. 10d) of the MG-sample that was heated in a DSC up to 700 °C.

### Cold spray coating

**Figure 11:**
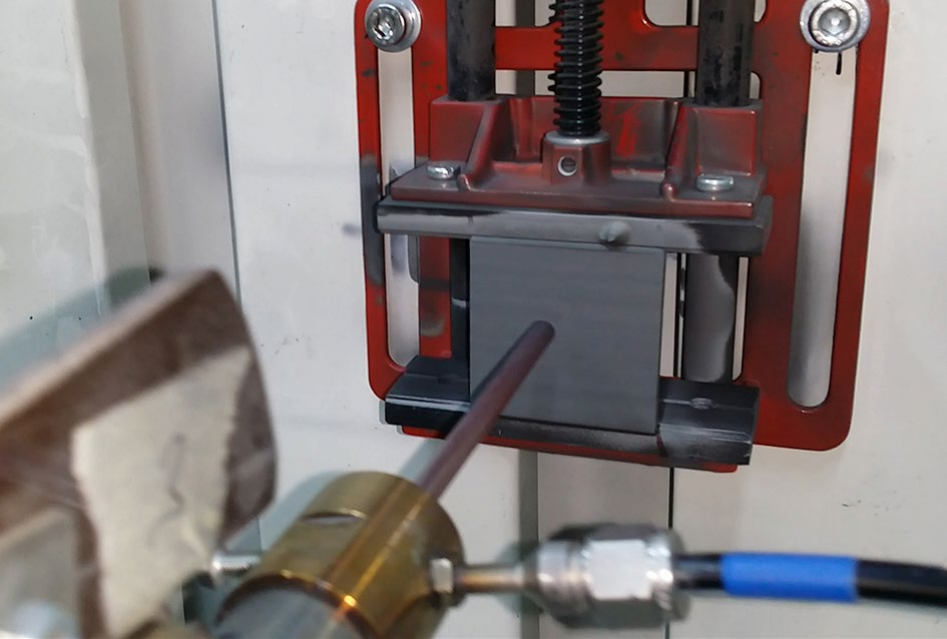
An onsite photo displays the cold spray coating procedure used to prepared MG coated/ SUS 304 at 550 °C for five continuous times.

Figure 11 displays a photo taken during the cold spray coating process that was carried out in the present work. In this study, the as-synthesized metallic glassy powder particles (taking Cu_50_Zr_20_Ni_30_, as a typical example), obtained after 50 h of MA time, were used as antibacterial feedstock materials for coating stainless steel sheets (SUS304) using a cold spraying technique. The cold spray approach was selected for the purpose of coating among the thermal spray family of techniques because it is the most effective approach in the thermal spray family, in which it can be used in coating of metallic metastable temperature sensitive material (such as amorphous and nanocrystalline phases) powders without obeying to phase transformations. This was the primary factor in the selection of this method. The cold spray process is taking place by utilizing high-velocity particles, which, upon impact with a substrate or previously deposited particles, convert the particle's kinetic energy into plastic deformation, strain, and heat^42^.

It is necessary for the kinetic energy of a particle as well as the momentum of each particle in the coating formation to be converted into other forms of energy through mechanisms such as plastic deformation (both substrate and particle interactions for the initial particles and particle–particle interactions as the coating formation builds), void consolidation, particle–particle rotation, strain, and ultimately heat^39^. Furthermore, if not all of the incoming kinetic energy is converted into heat and strain energy, the outcome is an elastic collision, which means that the particle will simply rebound after the impact. It has been pointed out that 90% of the applied impact energy on the particle/substrate materials is converted into local heat^40^. Additionally, when the impact stresses are applied, high plastic strain rates are achieved in the contact particle/substrate zone within a very short time^41,42^.

Plastic deformation is often thought of as a process for the dissipation of energy, or more specifically, as a source of heat in the interfacial area. However, the temperature increase in the interfacial region is typically not sufficient to produce interfacial melting or to significantly promote atomic interdiffusion. There are no publications that the authors are aware of that investigate the influence of the features of these metallic glassy powders on the bonding and deposition of powders that occur when cold spray method is used.

**Figure 12:**
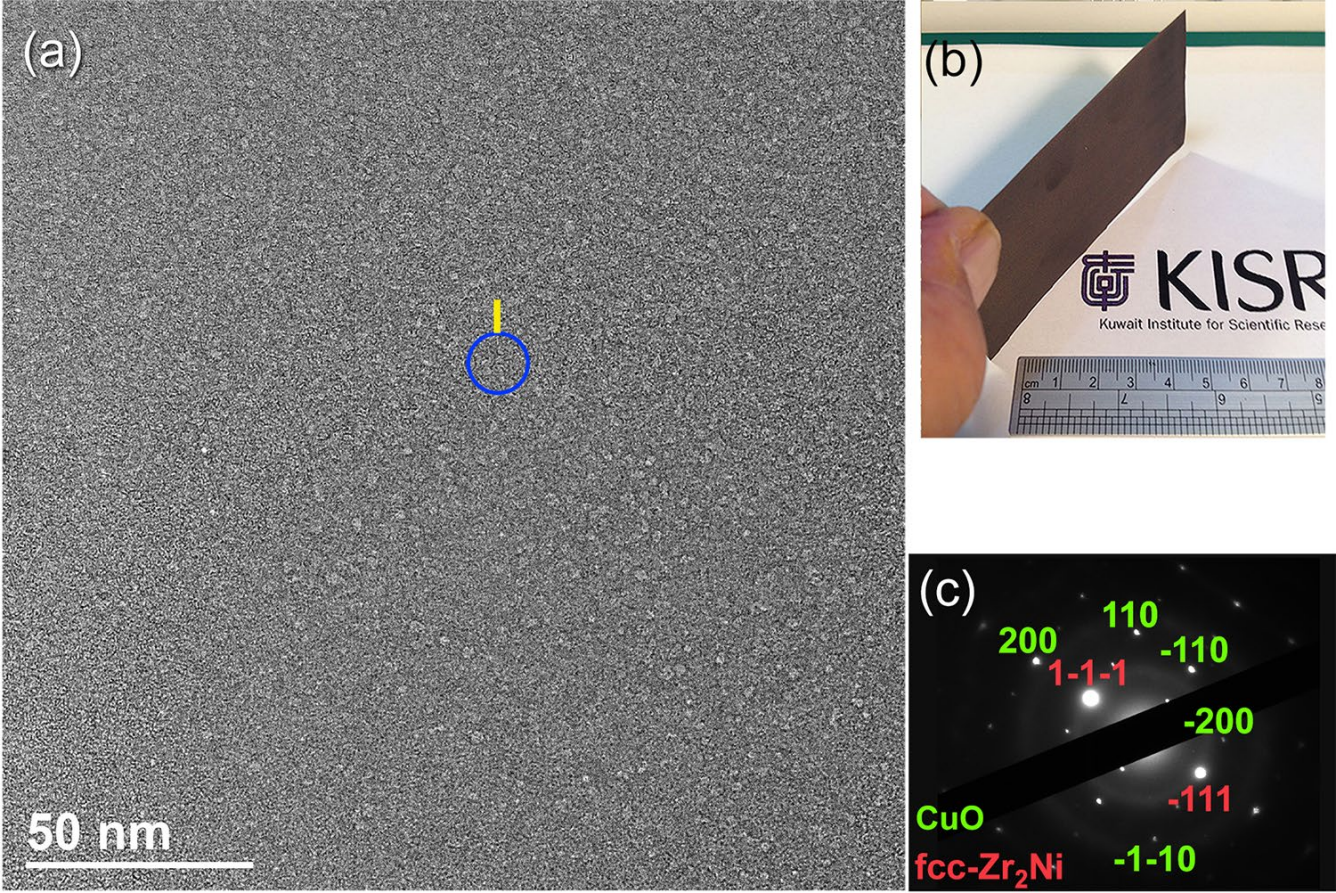
(**a**) FE-HRTEM image of MG powders that were coated on (**b**) SUS 304 substrate (inset of the figure). The indexed NBDP of the circular symbol shown in (**a**) is displayed in (**c**).

The BFI of MG Cu_50_Zr_20_Ni_30_ alloy powders can be seen in Fig. 12a, which was coated on a SUS 304 substrate (Figs. 11, 12b). It can be seen in the image that the coating powders have maintained their original amorphous structure since they have a delicate maze structure without any crystalline features or lattice defects. The image, on the other hand, suggested the presence of foreign phase(s), which was hinted by the nanoparticles that were incorporated into the matrix of the MG coating powders (Fig. 12a). Figure 12c depicts the indexed nanobeam diffraction pattern (NBDP) that is associated with zone I (Fig. 12a). As can be seen in Fig. 12c, the NBDP exhibited a weak halo diffuse pattern of an amorphous structure coexisted with sharp patches that corresponded to crystalline big-cube Zr_2_Ni metastable—plus tetragonal—CuO phases. When traveling from the nozzle of the gun under supersonic flow toward the SUS 304 in an open air, the formation of CuO may be attributed to oxidation of the powders. On the other hand, the formation of big-cube phase was realized to devitrification of the metallic glassy powders upon cold spray processing at 550 °C for 30 min.

An independent experiment was conducted in order to verify this potential mechanism for the formation of big cube Zr_2_Ni nanoparticles. During this experiment, the powders were sprayed from the gun in the direction of the SUS 304 substrate at 550 °C; however, they were removed from the SUS304 strip as soon as possible (~ 60 s) in order to illuminate the annealing effect on the powders. Another set of experiments was carried out, and in this trial, the powders were removed from the substrate after deposition for ~ 180 s.

**Figure 13:**
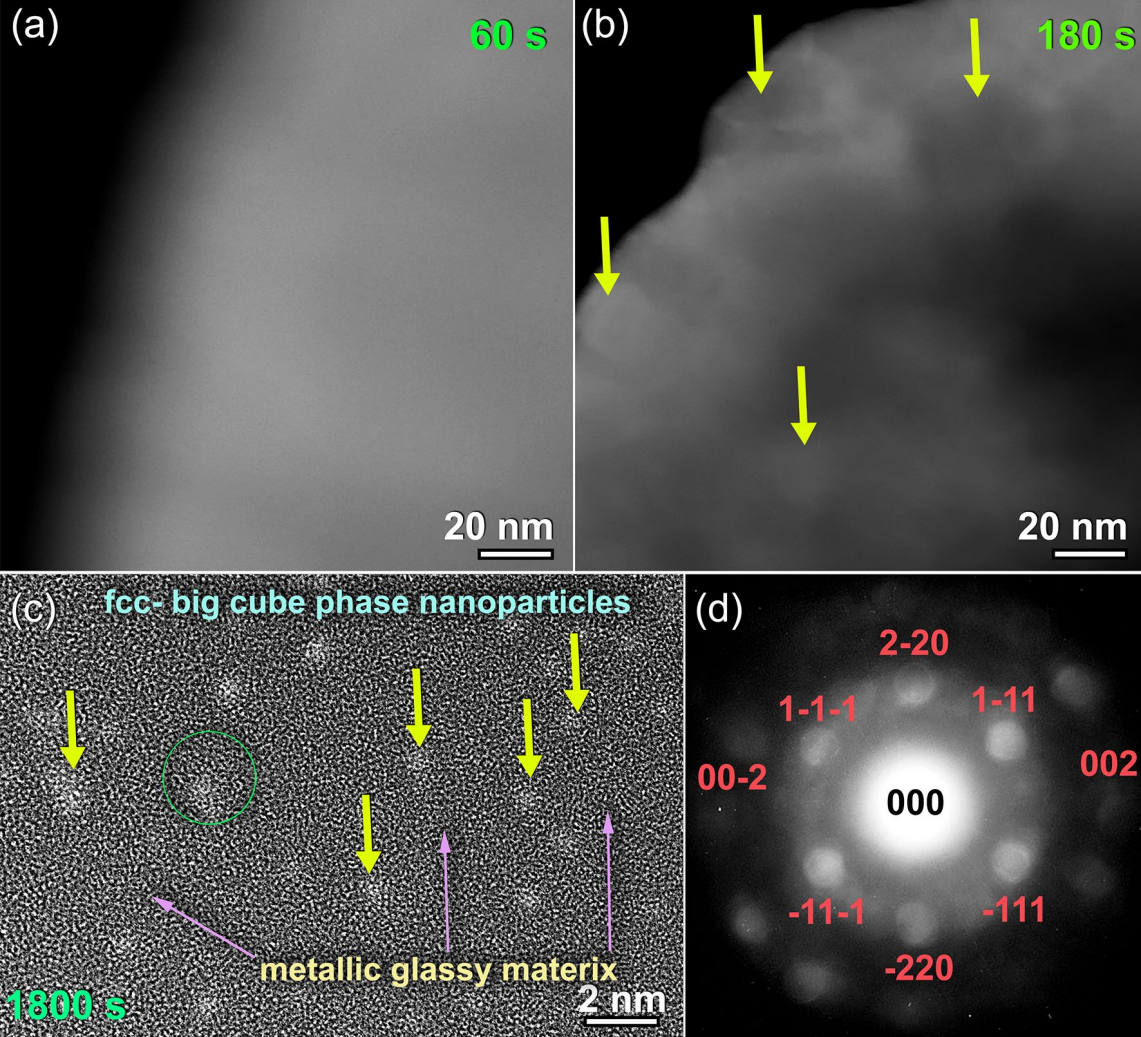
Scanning transmission electron microscope/dark field images (STEM/DFIs) of cold Cu_50_Zr_20_Ni_30_ MG powders deposited on SUS 304 °C for (**a**) 60 s, and (**b**) 180 s. The atomic resolution TEM image, and the related NBDP of the circular symbol indexed in Fig. 12a for the powders deposited on the substrate for 1800s are displayed in (**c**) and (**d**), respectively.

**Figure 14:**
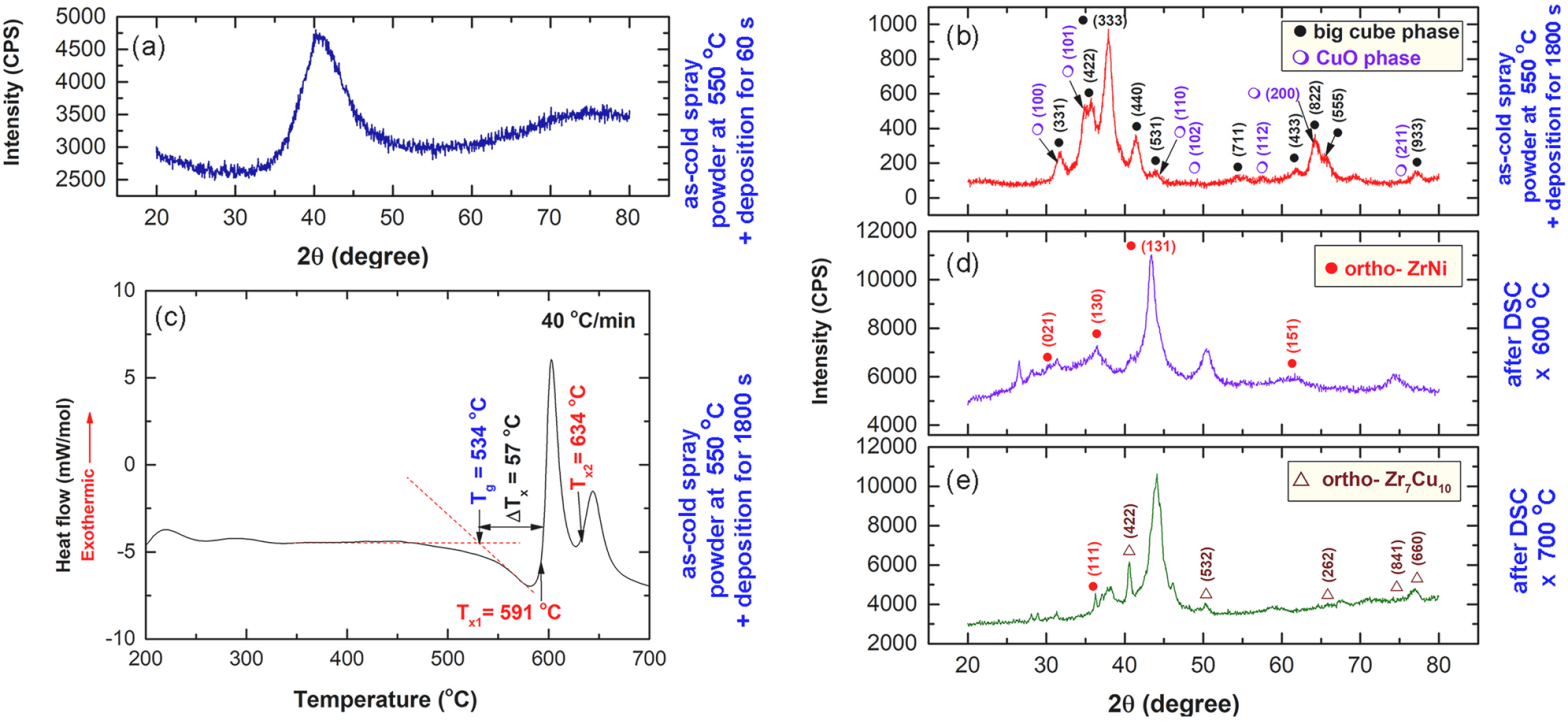
Effect of cold spray on the structure and thermal stability of MG Cu_50_Zr_30_Ni_20_ powders. XRD pattern of MG Cu_50_Zr_30_Ni_20_ powders obtained after 50 h of MA time are displayed in (**a**). The XRD pattern and corresponding DSC thermogram of the MA powders that were cold spray at 550 °C are displayed in (**b**) and (**c**), respectively. The XRD patterns of the cold sprayed MG powders heated up to 600 °C, and 700 °C, are displayed in (**d**) and (**e**), respectively.

Figure 13a,b show the dark field images (DFIs) obtained by a scanning transmission electron microscope (STEM) for the two sprayed materials that were deposited on a SUS 304 substrate for 60 and 180 s, respectively. The image of the powders deposited for 60 s had no morphological details and revealed featureless (Fig. 13a). This is also was confirmed by the XRD, which indicated that the general structure of these powders is amorphous, as suggested by the broad primary and secondary diffraction maxima displayed in Fig. 14a. These indicate the absence of precipitation of metastable/intermediate phase, where the powders maintain their original amorphous structure. In contrast, the powders sprayed at the same temperature (550 °C), but left on the substrate material for 180 s have shown precipitation of nanodimensional crystalline grain, as indexed by the arrows shown in Fig. 13b.

Figure 13c,d, respectively, depict the FE-HRTEM image and corresponding NBDP of zone I that is shown in Fig. 12a. During the cold spray procedure, which was repeated five times at 550 °C for 1800s, a significant volume fraction of nanocrystalline spherical grains were obtained, and these grains tended to be embedded into the metallic glassy matrix, as shown in Fig. 13c. This is indicated by the atomic resolution TEM image that was obtained. As suggested by the indexed NBDP, the NBDP was able to validate that these nano-spheres were connected to the big-cube form of Zr_2_Ni^43^ (Fig. 13d).

The analysis of the diffracted lines presented in Fig. 14b reveals that the deposited powders for 180 s are connected to Ti_2_Ni-structure^43^ (E9_3_ structure, space group Fd3m). The lattice constant, a_0_, of this crystalline phase was determined to be 1.2295 nm by using the principal diffracted line (3 3 3) in Fig. 14b.

Figure 14c shows the DSC curves of the powders after they were sprayed five times at 550 °C for 1800s. By comparing the scan of this sample with that of powders acquired after 50 h of MA time during which the crystallization process was carried out in a single step, we were able to determine that there was a significant difference between the two samples (Fig. 10c). It has come to attention that there has been a significant shift in the way crystallization operates as a result of the cold spray technique. This is implied by the change in the crystallization process, which takes place through two steps, as characterized by the two exothermic reactions that appeared at 591 °C and 634 °C, respectively, as shown in Fig. 14c. The crystallization processes, which was taken place through two steps has led to the formation of orthorhombic-phases of ZrNi, and Zr_7_Cu_10_, as evidenced by the XRD displayed in Fig. 14d,e, respectively.

**Figure 15:**
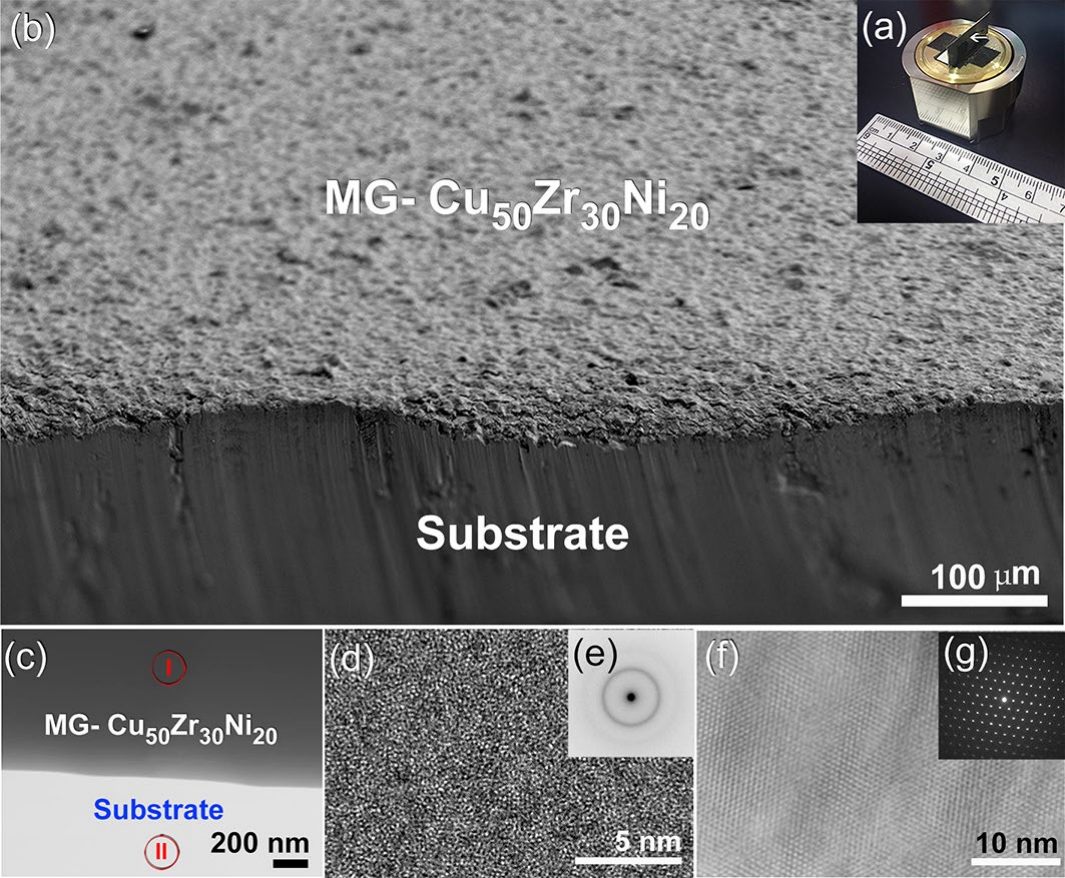
A typical MG-Cu_50_Zr_30_Ni_20_ coated/SUS304 sample, which was vertically mounted on Cu-SEM sample holder (**b**) is displayed in figure (**a**). The STEM micrograph for the ion polished samples is displayed in (**c**). In the meanwhile the FE-HRTEM of Zone I and Zone II indexed in (**c**) are displayed in (**d**) and (**f**) together with their NBDPs (**e**,**g**), respectively. The FE-SEM image of the plan view of this sample is shown in (**b**).

The typical MG-Cu_50_Zr_30_Ni_20_ coated/SUS304 sample, which was vertically mounted on Cu-SEM sample holder, is displayed in Fig. 15a. The FE-SEM image of the plan view of this sample is shown in Fig. 15b. As can be seen in Fig. 15b, the sample, which has a thickness of less than 22 µm, exhibited features of dense structure and a surface morphology that was relatively rough. It can be realized the absence of micro cracks at the interface between the coating- and substrate-materials, implying the formation of well bonded composite, as shown in Fig. 15b.

More detailed characterizations of the coating and substrate materials have been obtained by STEM technique. The plan view STEM image of the as-ion milled sample is displayed in Fig. 15c. This low magnification STEM image has indicated a good bonding beyond the nano level between the coating MG- Cu_50_Zr_30_Ni_20_ material and SUS304 substrate, as characterized by the absence of pores and cracks at the interface (Fig. 15c). The FE-HRTEM of regions I, and II indexed in Fig. 15c are shown in Fig. 15d,g, respectively. The corresponding image of zone I revealed noncrystalline structure with maze-like morphology, indicating the presence of an amorphous phase (Fig. 15d). Moreover, the NBDP (Fig. 15e) related to the image displayed in Fig. 15c revealed halo pattern of an amorphous structure. The FE-HRTEM image and corresponding NBDP related to zone II, which is located at the substrate material (SUS 304) are displayed in Fig. 15f,g, respectively. The sample, which revealed continuous staking faults displayed fringe-images related to austenitic SUS304 of zone axis < 110 > , as shown in Fig. 15f,g.

**Figure 16:**
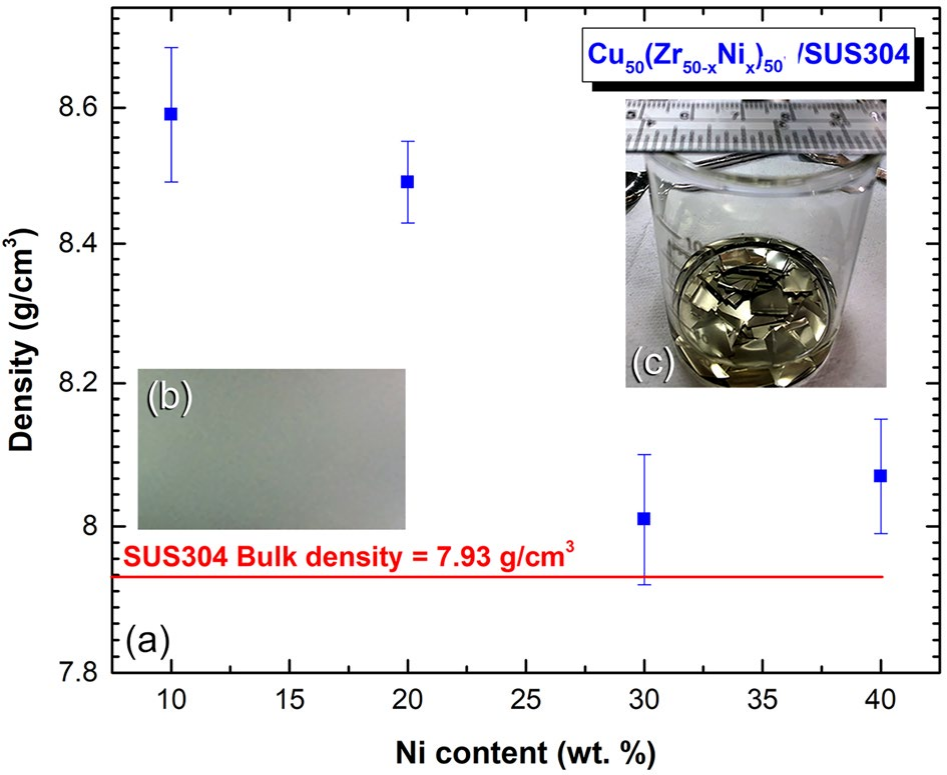
(**a**) Measured bulk density of MG-Cu_50_(Zr_50−x_Ni_x_)_50_ coated/SUS304. The SUS304 substrate before and after cold spray coating are displayed in (**b**) and (**c**), respectively.

Figure 16a shows the bulk density of MG-Cu_50_(Zr_50−x_Ni_x_)_50_, where x equals to 10, 20, 30, and 40 at.% coated SUS304. The density measurements were conducted at ambient temperature with pure toluene, using Archimedes’ approach. For the purpose of this investigation, the sheet of SUS304 substrate (Fig. 16b), which was coated with ~ 25 µm thickness MG-powders was divided into equals coupons of (1 cm × 1 cm), as displayed in Fig. 16c. The weight of each sample with different Ni concentrations, and their dimensions were measured preciously, to calculate the volume of the samples. This procedure were repeated three times, using three different samples. The density of pure SUS304 (before coating) was measured and found to be 7.93 g/cm^3^ (Fig. 16a). The density of the composite sample contains 40 at.% Zr was 8.56 g/cm^3^ (Fig. 16a). Increasing the Zr content to 20 at.% and 30 at.% led to a significant decrease in the density to be 8.46 and 8.06 g/cm^3^ respectively, as shown in Fig. 16a. MG-Cu_50_Zr_10_Ni_40_ coated/SUS304 had a bulk density of 8.03 g/cm^3^.

### Microbiological testing

**Figure 17:**
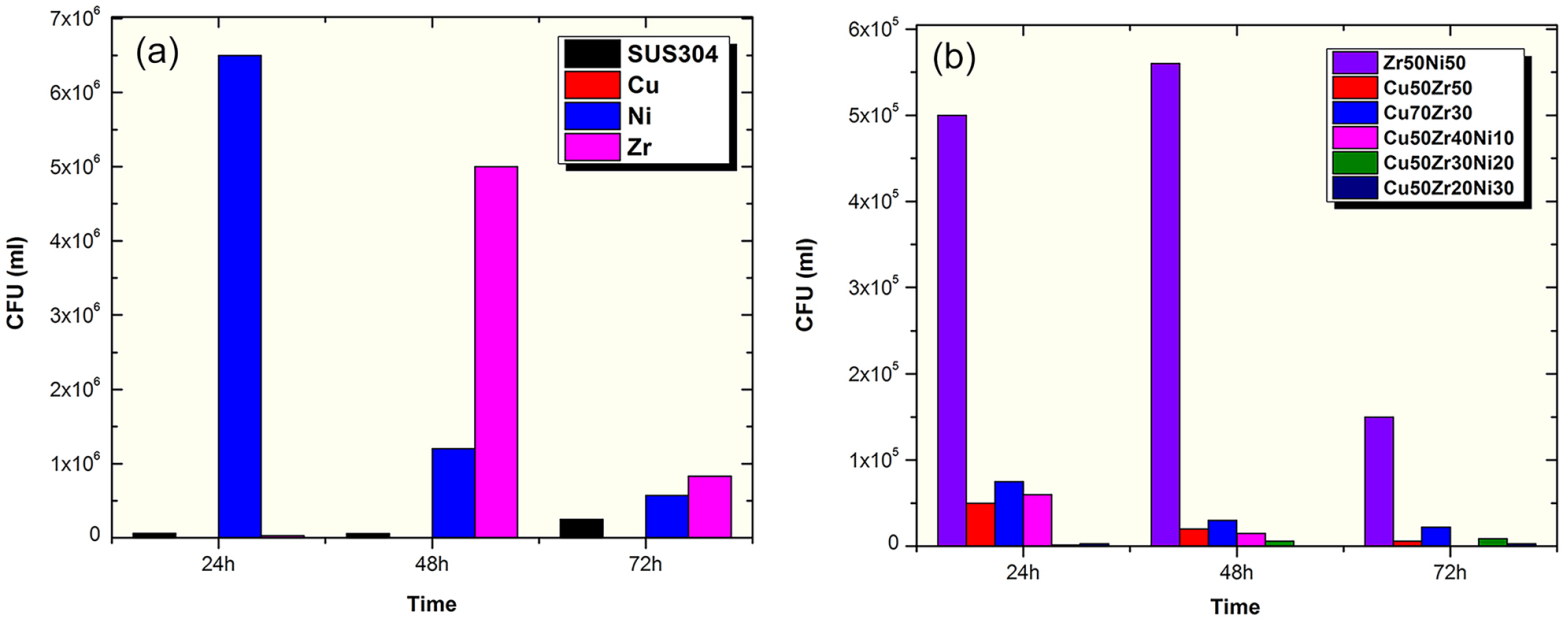
Inhibition of biofilm adhesion on (**a**) uncoated SUS304 coupons, and elemental Cu, Ni, and Zr metals, (**b**) binary Zr_50_Ni_50_, Cu_50_Zr_50_, and Cu_70_Zr_30_ systems. The results for inhibition of biofilm adhesion of selected ternary Cu_50_(Zr_50−x_Ni_x_) systems (x; 10, 20, 30 at.%) are displayed in (**b**). *E. coli* (ATCC 25922) biofilm viable counts were enumerated at different time points (24, 48 and 72 h) from tested coating alloy (13b) or control coated and non-coated coupons (13a). Biofilm formed by E.coli (ATCC 25922) was significantly inhibited by ternary systems (CuZrNi) at all times points tested in comparison to control coated coupons. Results represents mean ± SD of 3 independent experiments.

To evaluate the inhibitory effect of metallic glass coating/SUS304 to biofilm formation, biofilm formation on elemental metals (Cu, Zr, Ni), binary (Zr_50_Ni_50_, Cu_70_Zr_30_) and ternary (Cu_50_(Zr_50−x_Ni_x_)) systems in addition to a viability of cells released from the coated and non-coated coupons was investigated. Gram negative *Escherichia coli* (ATCC 25922) were selected as model bacteria. The inhibitory effect of coated surfaces was quantitively assisted by colony forming unit (CFU)/ml. Mean colony counts in both types of coupons are shown in Fig. 17.

Figure 17a shows the inhibitory effect of cold spray coated SUS304 substrate with elemental Cu, Ni, and Zr metals. In the meanwhile, the antibacterial behavior of cold spray coated SUS304 binary (Zr_50_Ni_50_, Cu_50_Zr_50_, and Cu_60_Zr_30_), and ternary (Cu_50_Zr_40_Ni_10_, Cu_50_Zr_30_Ni_20_, and Cu_50_Zr_20_Ni_30_) against *E. coli* (ATCC 25922) biofilm formation incubated for 24 h, 48 h and 72 h are displayed together In Fig. 17b. Although it was reported that the antimicrobial effect of copper ions is dose-dependent^23^, in our results (Fig. 17a), only pure Cu-100% nano-coating shows no growth. However, similar antimicrobial activity was also well obtained with only Cu-50% in case of Cu_50_Zr_40_Ni_10_ (no growth after 72 h), Cu_50_Zr_30_Ni_20_ (2.8 × 10^3^ CFU/ml after 72 h) and Cu_50_Zr_20_Ni_30_ (8 × 10^3^ CFU/ml after 72 h) which shows more than 95% biofilm inhibition in comparison to systems without Cu nano-coating (Zr_50_Ni_50_), as shown in Fig. 17b. Moreover, substrates coated with Cu_50_Zr_20_Ni_30_, Cu_50_Zr_30_Ni_20_ and Cu_50_Zr_40_Ni_10_ significantly inhibited colony formation of *E. coli* (Fig. 17b) by at least one log at all times in comparison to SUS304, Ni and Zr control alone (Fig. 17a). These results were statistically significant (p < 0.05, n = 3).

It well known that mature biofilm cells are more resistant to treatment with antimicrobial reagents than planktonic cells, classic treatment with antibiotic are usually not sufficiently to reduce biofilms formed on implant devises and the replacement of the implant is the only way so solve the problem^44–47^. Many studies demonstrated the antibiofilm inhibitory effect of Zr, Cu and Ni on coated surfaces. It has been reported that coated stainless steel with 304-Cu, 420-Cu and 317L-Cu metallic alloys showed strong antibacterial effect against biofilm formation due to a continuous release of Cu ions into the environment^48,49^. Moreover, the antibiofilm inhibitory effect of Zr has been reported by Al-Radha et al. who reported that pure zirconia showed significant effect against biofilm smooth surfaces^50^. Manicone et al.^51^, demonstrated that use of ZrO_2_ surfaces can significantly inhibit the adhesion of bacterial cells in dental implants^51^. It has been, pointed out that Zr-based bulk metallic glasses can inhibit biofilm formation up to 99.9%^52^. Meanwhile, other researchers demonstrated that surfaces coated with Zr–Cu–Ni–Al improve the efficacy of the coated SUS304 against biofilms formed by *E.coli* and *Staphylococcus aureus* (*S. aureus*)^53,54^.

The antibiofilm mode of action of Cu and the effect of releasing of Cu^2+^ has been widely reported^48–50^. The release of Cu^2+^ ions from metallic surfaces and the direct contact with bacterial cells are the main reasons for the Cu to be effective. Moreover, antimicrobial effect of Cu includes bacterial cell injuries, outer and inner cell membrane damage, oxidative damage due to production of reactive oxygen species (ROS), inhibition of enzyme production and nucleic acid degradation has been reported^49^.

It has been demonstrated that the combination of Cu, Zr and Ni can reduce the bacteria attachment up to 99%^53^. However, most studies showed the inhibition of biofilm formation after coating the surfaces with more than 90% Cu (Table 3), in this study we can achieve significant inhibition with only 50% Cu content. Our present results supports and suggest that Cu_50_Zr_20_Ni_30_, Cu_50_Zr_30_Ni_20_ and Cu_50_Zr_40_Ni_10_ coated materials (Fig. 17b) will be very effective against biofilm formation. Nevertheless, the only drawback of our present study is that one species of bacteria has been used as a model, which may not reflect in vivo environments, multi biofilm forming bacteria need to be investigated.

**Table 3:** Some researches from earlier studies presented several antibiofilm technologies that use distinct coating strategies.

Coated materials	Coating approach	Bacterial strain	Percentage inhibition	References
Elemental Cu	Ready made	*Acinetobacter calcoaceticus* *Stenotrophomonas maltophilia*	> 3.5 log CFU/cm^2^	Gomes et al.^55^
Cu_90_Ni_10_	Ready made	*Pseudomonas *sp.	< 10^3^ CFU/ml	Vanithakumari, et al.^56^
4 × Cu–TiO_2_	Arc melting	*Staphylococcus epidermidis*	< 10^4^ CFU/ml	Mauerer, et al.^57^
Steriall^®^ copper alloys Cu 90 and 70%	Ready made	Environmental bacteria	2.0 CFU/cm^2^	Colin, et al.^58^
Ti—5 wt% Cu	Arc melting	*S. aureus* *E. coli*	3.5 × 10^3^ CFU/ml (24 h)	Liu et al.^59^
Ti_76.2_Zr_3.4_Cu_29.5_	Arc melting	*S. aureus* *E. coli*	10^3^ CFU/ml	Kolawole et al.^60^
Co_0.4_FeCr_0.9_Cu_*x*_ (*x* = 0.3 and 0.5) Cu-HEAs	Arc melting	*S. aureus* *E. coli*	< 10^3^ CFU/ml	Ren et al.^61^
Cu_50_Ti_40_Ni_10_	Cold spray	*E. coli*	< 10^3^ CFU/ml	El-Eskandarany and Aldhameer^44^
Cu_50_Zr_40_Ni_10_	Cold spray	*E. coli*	< 10^3^ CFU/ml	Our present study

## Conclusions

Based on the present study, ternary Cu_50_(Zr_50−x_Ni_x_) metallic glassy systems (x; 10, 20, 30, and 40 at.%) were synthesized from elemental powders, using low energy ball milling technique. The end-product obtained after 50 h revealed high thermal stability, as indexed by high T_g_ (447–526 °C), and T_x_ (526–612 °C), with large ΔT_x_. For the purpose of the present work, cold spray coating approach was used to fabricate Cu_50_(Zr_50−x_Ni_x_) metallic glassy coated/SUS 304 sheets at temperature being just above the T_g_. Due to the heat generated during plastic deformation of SUS 304 substrate taken place during cold spraying process, significant volume fractions of metallic glassy coating powders devitrificated into a big cube Zr_2_Ni metastable phase. According to the results produced in this study, ternary systems (CuZrNi) were the most effective coating metals for inhibition of *E. coli* bacterial cell adhesion in comparison to other systems investigated. Moreover, CuZrNi systems showed prolonged antibiofilm effect up to 72 h. Although CuZrNi metallic alloys demonstrate the best antibiofilm inhibitory effect against the model *E. coli* species, it should not consider as the only problem solver of biofilm formed on surfaces. It has been reported that bacterial strains can develop a resistance pathways to metals similar to resistance developed to antibiotic treatments. Thus, overcome resistance of biofilm to metal should be further studied.

## Experimental procedure

### Materials and methods

#### Preparations of Cu-based metallic glassy alloy powders by mechanical alloying

Powders of pure metallic alloying elements such as Cu, Ti, Zr, Nb, and Ni (with purity more than 99.9 wt% and diameter less than 20 µm) were employed as starting reactant. The powders of the system listed in Table 1 were balanced to give the average nominal composition of the starting charge for Cu-based binary, ternary and multicomponent system (Table 1), and then mixed in a glove box (UNILAB Pro Glove Box Workstation, mBRAUN, Germany) filled with helium gas to obtain the desired composition. Then after, a certain quantity (150 g) of the powders for the desired system were charged into an Cr-steel vial (1000 ml in capacity) and sealed along with 100 Cr-steel balls (14 mm in diameter). The weight ratio of the balls-to-powder was 36:1. The MA process was started by mounting the vial on a roller mill (RM20) provided by Zoz GmbH, Germany, operated at room temperature with a rotation speed of 235 rpm. The progress of the solid-state reaction was monitored by interrupting the MA process after selected ball milling time, where the vial was opened in the glove box to take a represented sample. All samples were then characterized by different analysis.

#### Fabrication of Cu-based metallic glassy powders coated/SUS304 composites by cold spray process

In recognition of the way that the powders were noncrystalline (amorphous) when they were first synthesized, it was anticipated that they would crystallize into a stable (crystalline) phase when heated above their crystallization temperature. Given that the goal of this research is to determine if metallic glassy Cu-based metallic glassy alloy powders impact the growth of biofilms, it is critical that the glassy phase be maintained throughout the spraying process. A cold spraying procedure was used to cover both sides of SUS304 sheets as a result of this. As a substrate metal, stainless steel (SUS304) sheets were utilized, which were first cleaned with acetone and ethanol and then dried in an oven at 150 °C for 1 h. Before the coating process began, the surface of the substrate was prepared with alumina blasting at ambient temperature. It is important to note that, unlike thermal spray combustion-based approaches, the cold spray approach is accomplished at low temperatures (in the range of 100–900 °C), which is far lower than the melting points of the feedstock powders. In the present work, the cold spraying process was initiated at a low temperature (400 °C) with a supersonic jet processed at a very high velocity (1200 m/s).

### Materials characterizations

#### General structure

Using equipment from RIGAKU-SMARTLAB 9 kW, X-ray diffraction was used to evaluate the general structural changes that occurred as a result of ball milling Cu-based master alloys. Using CuKα radiation with a wavelength of 0.15418 nm and an operating voltage of 45 kV 200 mA, all of the samples were evaluated at a speed of 2θ/min through a continuous 2/θ scan mode. The detector utilized was a high-speed 1D X-ray detector called D/teX Ultra 1D mode (D/teX) with Ni Filter. The diffraction patterns were acquired across a range of 20° to 80° in 2θ, with a step size of 0.02/2 and a duration of 1 s/step for each stage in the process. The XRD was produced as a consequence of constructive and destructive interference brought about by the scattering of X-rays from atoms arranged in a regular array. Diffraction lines appeared at angles that were consistent with Bragg's method.

Using JEOL microscopes of the JEOL 2000F model with a resolution of 0.17 nm and operating at a voltage of 200 kV, a field emission high-resolution transmission electron microscope (FE-HRTEM) that was equipped with energy-dispersive X-ray spectroscopy (EDS) was used to examine powder samples of as-synthesized materials. After dissolving the sample powders in ethanol, a few drops of the resulting solution were placed on a copper (Cu)-microgrid and allowed to dry in a vacuum. After that, the microgid was installed onto the TEM transfer rod before being transferred to the vacuum sample chamber of the TEM. EDS was utilized to do elemental analysis on the micrographs that were acquired for the bright field image (BFI), dark field image (DFI), and selected area electron diffraction patterns (SADPs). Details of these measurements but for a different system are described elsewhere^44^.

#### Morphology and local analysis

Field emission scanning electron microscopy (FESEM/EDS), using JEOL: JSM-7800F, operated at an acceleration power of 15 kV, was used to investigate the morphological characterizations of the samples and their elemental compositions. The powder samples were placed on double-sided adhesive carbon tape and placed on a Cu-sample holder. The samples prevented any possible charging in the image and kept the powder steady. The samples were inserted into the FE-SEM chamber for analysis. The concentrations of the metallic alloying elements in the as-ball milled powders were determined by both of TEM/EDS, and SEM/EDS techniques^44^.

#### Thermal stabilities

Shimadzu Thermal Analysis System/TA-60WS, using differential scanning calorimeter (DSC) was employed to investigate the thermal stability of the as-ball milled powders, indexed by the transition glass temperature (T_g_), and crystallization temperature (T_x_), using a heating rate of 40 °C/min.

#### Bacterial strain and biofilm growth conditions

Escherichia coli (ATCC 25922) was used as a test organism. Biofilms were grown according to our previous work^25^. Sterile monocoated systems (Cu, Zr, Ni), binary systems (ZrNi, CuZr) and ternary systems (CuZrNi) triplicate coupons (22-mm^2^) were positioned vertically in 50-ml conical tubes with 6 ml pre-warmed BHI (Brain Heart Infusion). 100 μl 0.5 McFarland standard suspensions (equivalent to 1.5 × 108 CFU ml^−1^) of a 24 h culture Planktonic cells were added to each tube. Bacterial inoculum preparations, overnight bacterial culture was centrifuge (8000*g*, 10 min) to produce cell pellet, bacterial cells were then washed with deionized water followed by resuspension in BHI and optical density was set to 108 CFU/ml. Tubes was then incubated on a shaker to allow biofilm to form. Triplicate coated coupons was removed at each time point (24, 48, 72 h) and then rinsed with phosphate buffer solution (PBS) to remove non-adherent bacterial cells. Coated coupons were then transfer to fresh tube with 6 ml BHI and vortex for 1 min at maximum speed. For viable count, suspension result after vortexing were then serially diluted in PBS and plated on nutrient agar (NA) viable bacteria were then enumerated.

## Data Availability

The datasets used and/or analysed during the current study available from the corresponding author on reasonable request.

## References

[1] El-Eskandarany. M. S. *Mechanical Alloying: Energy Storage, Protective Coatings, and Medical Applications*, 3rd ed. (Elsevier, 2020).

[2] Aizikovich SM, Altenbach H (2020). Modeling, Synthesis and Fracture of Advanced Materials for Industrial and Medical Applications (Advanced Structured Materials).

[3] Bin SJB, Fong KS, Chua BW, Gupta M (2022). Mg-based bulk metallic glasses: A review of recent developments. J. Magnes. Alloys.

[4] Klement W, Willens R, Duwez P (1960). Non-crystalline structure in solidified gold-silicon alloys. Nature.

[5] Greer AL (2015). New horizons for glass formation and stability. Nat. Mater..

[6] Greer AL (2009). Metallic glasses…on the threshold. Mater. Today.

[7] Sun Y, Concustell A, Greer AL (2016). Thermomechanical processing of metallic glasses: extending the range of the glassy state. Nat. Rev. Mater..

[8] Suryanarayana C (2019). Mechanical alloying: A novel technique to synthesize advanced materials. Research.

[9] Koch CC, Cavin OB, McKamey CG, Scarbrough JO (1983). Preparation of amorphous Ni_60_Nb_40_ by mechanical alloying. Appl. Phys. Lett..

[10] El-Eskandarany MS, Saida J, Inoue A (2002). Amorphization and crystallization behaviors of glassy Zr_70_Pd_30_ alloys prepared by different techniques. Acta Mater..

[11] El-Eskandarany MS, Saida J, Inoue A (2003). Structural and calorimetric evolutions of mechanically-induced solid-state devitrificated Zr_70_Ni_25_Al_15_ glassy alloy powder. Acta Mater..

[12] El-Eskandarany MS, Saida J, Inoue A (2003). Room-temperature mechanically induced solid state devitrifications of glassy Zr_65_Al_7.5_Ni_10_Cu_12.5_Pd_5_ alloy powders. Acta Mater..

[13] Schwarz RB, Koch CC (1986). Formation of amorphous alloys by the mechanical alloying of crystalline powders of pure metals and powders of intermetallics. Appl. Phys. Lett..

[14] Wang B (2022). Mechanical alloying derived SiBCN-Ta_4_HfC_5_ composite ceramics: Study on amorphous tansformation mechanism. J. Non-Cryst. Solids.

[15] Avar B (2021). Structural stability of mechanically alloyed amorphous (FeCoNi)_70_Ti_10_B_20_ under high-temperature and high-pressure. J. Alloy. Compd..

[16] El-Eskandarany MS, Aoki K, Suzuki K (1992). Thermally assisted solid state amorphization of rod milled Al_50_Nb_50_ alloy. J. Appl. Phys..

[17] Liu L (1993). Atomic short-range order of amorphous Ta–Cu alloys prepared by mechanical alloying. Acta Phys. Sin..

[18] El-Eskandarany MS (1996). Thermally assisted and mechanically driven solid-state reactions for formation of amorphous AI_33_Ta_67_ alloy powders. Metall. Mater. Trans. A.

[19] El-Eskandarany MS, Aoki K, Suzuki K (1992). Calorimetric and morphological studies of mechanically alloyed Al-50 at.% transition metal prepared by the rod milling technique. J. Appl. Phys..

[20] El-Eskandarany MS, Sumiyama K, Suzuki K (2003). Crystalline-to-amorphous phase transformation in mechanically alloyed Fe_50_ powders. Acta Mater..

[21] Schaaf P, Rixecker G, Yang E (1994). Study of nanocrystalline and amorphous powders prepared by mechanical alloying. Hyperfine Interact..

[22] Zhang M, Sun J, Wang Y, Yu M, Liu F, Ding G, Zhao X, Liu L (2021). Preparation of stable and durable superhydrophobic surface on Zr-based bulk metallic glass. Colloids Surf. A Physicochem. Eng. Asp..

[23] Kreve S, dos Reis AC (2020). Bacterial adhesion to biomaterials: What regulates this attachment? A review. Jpn. Dent. Sci. Rev..

[24] Aldhameer A, El-Eskandarany MS, Kishk M, Alajmi F, Banyan M (2022). Mechanical alloying integrated with cold spray coating for fabrication Cu_50_(Ti_50__−__x_ Ni_x_), x; 10, 20, 30, and 40 at.% antibiofilm metallic glass coated/SUS304 sheets. Nanomaterials.

[25] El-Eskandrany MS, Al-Azmi A (2016). Potential applications of cold sprayed Cu_50_Ti_20_Ni_30_ metallic glassy alloy powders for antibacterial protective coating in medical and food sectors. J. Mech. Behav. Biomed. Mater..

[26] Escobar A, Muzzio N, Moya SE (2020). Antibacterial layer-by-layer coatings for medical implants. Pharmaceutics.

[27] Hartl H (2022). Antimicrobial adhesive films by plasma-enabled polymerization of m-cresol. Sci. Rep..

[28] Geng H (2016). Antibacterial ability and hemocompatibility of graphene functionalized germanium. Sci. Rep..

[29] Dunseath O (2019). Studies of Black Diamond as an antibacterial surface for Gram Negative bacteria: The interplay between chemical and mechanical bactericidal activity. Sci. Rep..

[30] Buchegger S (2019). Smart antimicrobial efficacy employing pH-sensitive ZnO-doped diamond-like carbon coatings. Sci. Rep..

[31] Olmo JA-D, Ruiz-Rubio L, Pérez-Alvarez L, Sáez-Martínez V, Vilas-Vilela JL (2020). Antibacterial coatings for improving the performance of biomaterials. Coatings.

[32] Bharadishettar N, Bhat KU, Panemangalore D (2021). Coating technologies for copper based antimicrobial active surfaces: A perspective review. Metals.

[33] Donlan RM (2001). Biofilm formation: A clinically relevant microbiological process. Clin. Infect. Dis..

[34] Chambers LD, Stokes KR, Walsh FC, Wood RJK (2006). Modern approaches to marine antifouling coatings. Surf. Coat. Technol..

[35] Hickok NJ, Shapiro IM (2012). Immobilized antibiotics to prevent orthopaedic implant infections. Adv. Drug Deliv. Rev..

[36] Lemire JA, Harrison JJ, Turner RJ (2013). Antimicrobial activity of metals: Mechanisms, molecular targets and applications. Nat. Rev. Microbiol..

[37] Ren J, Han P, Wei H, Jia L (2014). Fouling-resistant behavior of silver nanoparticle-modified surfaces against the bioadhesion of microalgae. ACS Appl. Mater. Interfaces..

[38] Wang X (2022). Corrosion and antimicrobial behavior of stainless steel prepared by one-step electrodeposition of silver at the grain boundaries. Surf. Coat. Technol..

[39] Raletz F, Vardelle M, Ezo’o G (2006). Critical particle velocity under cold spray conditions. Surf. Coat. Technol..

[40] Grigoriev S, Okunkova A, Sova A, Bertrand P, Smurov I (2015). Cold spraying: From process fundamentals towards advanced applications. Surf. Coat. Technol..

[41] Grujicic M, Saylor JR, Beasley DE, DeRosset W, Helfritch D (2003). Computational analysis of the interfacial bonding between feed-powder particles and the substrate in the cold-gas dynamic-spray process. Appl. Surf. Sci..

[42] El-Eskandarany MS, Ali N, Banyan M, Al-Ajmi F (2021). Cold gas-dynamic spray for catalyzation of plastically deformed Mg-strips with Ni powder. Nanomaterials.

[43] Altounian Z, Batalla E, Strom-Olsen JO (1987). The influence of oxygen and other impurities on the crystallization of NiZr_2_ and related metallic glasses. J. Appl. Phys..

[44] El-Eskanadrany, M.S. & Al-Azmi, A. Metallic Glassy Alloy Powders for Antibacterial Coating. US 9,609,874 B1 (2017). 10.1016/j.jmbbm.2015.11.030 26703232

[45] Pan CH, Zhou ZB, Yu XW (2018). Coatings as the useful drug delivery system for the prevention of implant-related infections. J. Orthop. Surg. Res..

[46] Donlan RM, Costerton JW (2002). Biofilms: Survival mechanisms of clinically relevant microorganisms. Clin. Microbiol. Rev..

[47] Wei T, Yu Q, Chen H (2019). Responsive and synergistic antibacterial coatings: Fighting against bacteria in a smart and effective way. Adv. Healthc. Mater..

[48] Jung WK, Koo HC, Kim KW, Shin S, Kim SH, Park YH (2010). Antibacterial activity and mechanism of action of the silver ion in *Staphylococcus aureus* and *Escherichia coli*. Appl. Surf. Sci..

[49] Mei L, Zheng M, Ye Z (2015). Toward a molecular understanding of the antibacterial mechanism of copper-bearing titanium alloys against *Staphylococcus aureus*. Adv. Healthc. Mater..

[50] Al-Radha ASD, Dymock D, Younes C, O’Sullivan D (2012). Surface properties of titanium and zirconia dental implant materials and their effect on bacterial adhesion. J. Dent..

[51] Manicone PF, Iommetti PR, Raffaelli L (2007). An overview of zirconia ceramics: Basic properties and clinical applications. J. Dent..

[52] Chu J-H, Lee J, Chang C-C, Chan Y-C, Liou M-L, Lee J-W, Jang JS-C, Duh J-G (2014). Antimicrobial characteristics in Cu-containing Zr-based thin film metallic glass. Surf. Coat. Technol..

[53] Secinti M, Ayten G, Kahilogullari G, Kaygusuz HC, Ugur A (2008). Antibacterial effects of electrically activated vertebral implants. J. Clin. Neurosci..

[54] Han K, Jiang H, Wang Y, Qiang J, Yu C (2021). Antimicrobial Zr-based bulk metallic glasses for surgical devices applications. J. Non-Cryst. Solids..

[55] Gomes IB, Simões LC, Simões M (2019). The role of surface copper content on biofilm formation by drinking water bacteria. RSC Adv..

[56] Vanithakumari SC, Yadavalli P, George RP, Mallika C, Kamachi Mudali U (2018). Development of hydrophobic cupronickel surface with biofouling resistance by sandblasting. Surf. Coat. Technol..

[57] Mauerer A, Stenglein S, Schulz-Drost S, Schörner C, Taylor D, Krinner S, Heidenau F, Adler W, Forst R (2017). Antibacterial effect of a 4x Cu-TiO_2_ coating simulating acute periprosthetic infection—An animal model. Molecules.

[58] Colin M, Klingelschmitt F, Charpentier E, Josse J, Kanagaratnam L, De Champs C, Gangloff SC (2018). Copper alloy touch surfaces in healthcare facilities: An effective solution to prevent bacterial spreading. Materials.

[59] Liu R, Tang Y, Zeng L, Zhao Y, Ma Z, Sun Z, Xiang L, Ren L, Yang K (2018). In vitro and in vivo studies of anti-bacterial copper-bearing titanium alloy for dental application. Dent. Mater..

[60] Kolawole SK, Ren L, Siddiqui MA, Ullah I, Wang H, Zhang S, Zhang J, Yang K (2022). Optimized mechanical properties, corrosion resistance and bactericidal ability of Ti-15Zr-xCu biomedical alloys during aging treatment. Acta Metall. Sin. (Engl. Lett.).

[61] Ren G, Huang L, Hu K, Li T, Lu Y, Qiao D, Zhang H, Xu D, Wang T, Li T, Liaw PK (2022). Enhanced antibacterial behavior of a novel Cu-bearing high-entropy alloy. J. Mater. Sci. Technol..

